# Embryos, Clones, and Stem Cells: A Scientific Primer

**DOI:** 10.1100/tsw.2004.121

**Published:** 2004-08-18

**Authors:** Kenyon S. Tweedell

**Affiliations:** Department of Biological Sciences, University of Notre Dame, Notre Dame, IN 46556, USA

**Keywords:** embryos, nuclear clones, chimeras, differentiation, transgenic, neural crest, cell regeneration, transdifferentiation, clones, embryonic and adult stem cells, somatic cell nuclear transplantation, reproductive cloning

## Abstract

This article is intended to give the nonspecialist an insight into the nuances of “clones”, cloning, and stem cells. It distinguishes embryonic and adult stem cells, their normal function in the organism, their origin, and how they are recovered to produce stem cell lines in culture. As background, the fundamental processes of embryo development are reviewed and defined, since the manipulation of stem cell lines into desired specialized cells employs many of the same events. Stem cells are defined and characterized and shown how they function in the intact organism during early development and later during cell regeneration in the adult. The complexity of stem cell recovery and their manipulation into specific cells and tissue is illustrated by reviewing current experimentation on both embryonic and adult stem cells in animals and limited research on human stem cell lines. The current and projected use of stem cells for human diseases and repair, along with the expanding methodology for the recovery of human embryonic stem cells, is described. An assessment on the use of human embryonic stem cells is considered from ethical, legal, religious, and political viewpoints.

## EMBRYONIC STEM CELLS

### The Marvelous Potential of the Egg and Embryo

A freedom peculiar to the embryo lies in its unlimited potential to produce all of the different cells, tissues, and organs found in the developing embryo and adult. The human embryo begins development after activation of the egg (oocyte) by the sperm in the fertilization process[1,2]. Prior to this, the oocyte develops into a mature egg according to a specific plan encoded in the chromosomes of the maternal nucleus. Once fertilized, the activated egg cell has a new nucleus, derived from a composite of the sperm and egg chromosomes, that will be duplicated at the time of cell division into each succeeding cell. Through the instructions found in the DNA of the genes contained in each cell's chromosomes, the entire catalog of genes (genome) can be called on. A specific gene or combination of genes in the nucleus can be instructed through interaction with molecules stored in the egg cell plasm (cytoplasm) that surrounds the nucleus. The fertilized egg (zygote) has the inherent ability to form all the transitory and definitive cells in the tissues and organs of the future adult organism of that species. These include both the precursors (primordial germ cells [PGCs]) of future eggs or sperm and each kind of specific body cell (somatic cell) found at each subsequent stage of development and in the adult; thus the zygote is totipotent, i.e., able to form a complete embryo.

## Early Embryonic Development

A direct outcome of fertilization is the division of the fertilized egg into two cells accompanied by duplication of the new nucleus formed by the male and female germ cells. Generally, each of these cells (blastomeres) will divide and yield four cells, each in turn dividing to produce eight cells, etc. In the human embryo, the first activation of the combined genome, i.e., male and female, is between the four and eight cell stages[3] when the new embryonic genome is expressed. Each of these cells possesses a complete copy of the nucleus and an isolated portion of the egg cell cytoplasm. Depending on the species, the successive cells may begin to lose their inherent developmental versatility yet still possess limited developmental potential. For example, a particular blastomere may no longer be able to produce germ cells, but may still have the potential to form a diverse number of different cell types.

Early in embryonic development, there are recognizable stages that are common to most higher animals and human embryos (Fig. 1):

1. Early division (cleavage): two to sixteen cells
2. Cell cluster (morula): a tight or loosely assembled ball of about sixteen cells
3. Blastula stage: often a two-layered sphere of cells

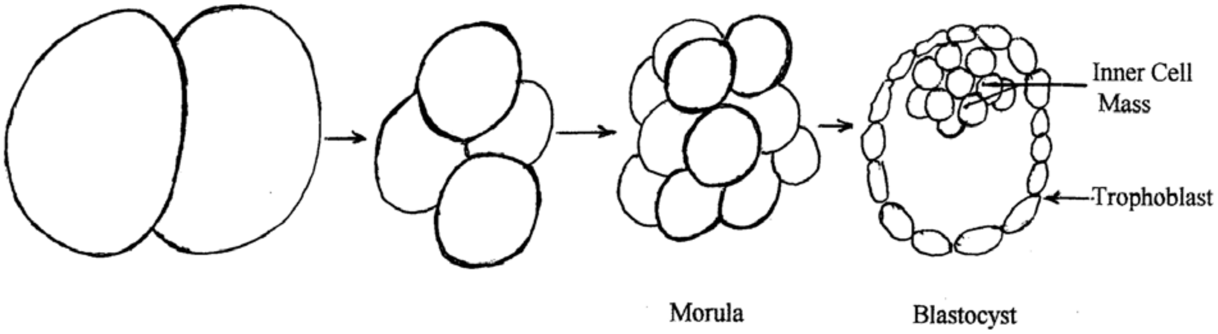

**FIGURE 1**

In mammals, the loosely knit early cleavage cells are held together by a gel-like layer. In addition, the blastula is modified for its existence in the uterus as a *blastocyst* stage that forms in humans about 4 days after fertilization. The blastocyst stage is unique since it develops an outer extraembryonic layer, the trophoblast layer, that later becomes membranes surrounding the embryo and a group of formative cells called the inner cell mass (ICM). The ICM is exclusively the precursor of the embryo. Between day 4 and day 5 postfertilization, the ICM develops two cell layers in an area known as the embryonic disc and is now regarded as a bilaminar embryo. Subsequently, the blastocyst emerges from the translucent gel layer and up to this point, the embryonic stages collectively have been considered by embryologists to be postfertilization embryonic stages or preimplantation embryos. It has been proposed by some that these early embryonic stages in human development (zygote, cleavage, and early blastocyst) should be considered as pre-embryos[4,5], however, O'Rahilly and Muller[2] oppose this definition. After the blastocyst emerges, the trophoblast attaches to and begins to penetrate the inner tissue wall of the uterus about 6 to 7 days after fertilization in a process of implantation. In normal events, the enclosed ICM will continue development into an embryo, but only after implantation of the entire blastocyst (conceptus) into the uterus. The outer trophoblast layer forms subsidiary embryonic membranes and contributes to part of the placenta whose main function is the care of the embryo. While this is going on, a profound change takes place in the embryo as the first of three embryonic germ layers (an inner **endoderm** and outer **ectoderm**) are established. This is an evolving process and when the third embryonic germ layer (the **mesoderm)** forms, the embryo becomes the *gastrula* — a stage from which all the specialized cells and tissues can be traced. This stage is a comparable period in all vertebrates, but its formation is unique to each species. Simultaneous with implantation, the gastrula embryo and subsequent stages of development are known as postimplantation embryos. In humans, up to the end of 8 weeks, the stages are designated as embryos; after 8 weeks until full term they are referred to as a fetus.

## *Process of Differentiation*

A property of early embryonic cell division that is usually manifested after the blastocyst stage, and a corollary to changes in their developmental potential, is that different cells or groups of cells will become more specialized in their appearance and/or function as development ensues. This process is known as embryonic differentiation and relates to stem cell differentiation since the same events and other controlling factors occur in each phenomenon. As cell specialization progresses, some cells are designated to form specific tissue precursors. Each of these associated groups of cells is derived from one of three embryonic germ layers whose identity is shared as recognizable tissue derivatives in all vertebrates. One layer, the ectoderm, will form the outer body covering (epidermis) and a specialized precursor of the brain, spinal cord, and nerves (neural ectoderm). A second embryonic layer, the mesoderm, is destined to become supporting tissues (bone, connective tissue, and muscle) and the third, endoderm, becomes the internal lining of digestive and respiratory organs. Differentiation of a primitive site in each germ layer domain is often a step-wise process. Certain cells become committed to a particular pathway and progress along specific steps leading to a unique form or function, such as the ability to contract in a tubular muscle cell. These steps are predictable and are often observed in the living embryo (*in vivo*) after staining with dyes or by the use of antibodies tagged with various compounds to reveal their presence on the cell. Differentiation of the cell is a process that starts with the formation of heterogenous cell types that are constantly changing in response to the cells in their microenvironment. Once adjacent cells are different, they each respond to their immediate neighboring cells in separate ways. Since the cell environment is also in flux, this involves a dynamic interaction of each new cell with its surroundings.

Differentiation in the early embryo is related to activation of certain nuclear genes found in the entire genome provided by the sperm and the egg. A basic tenant of gene activation or gene regulation in early development is that there is a "genomic equivalence among differentiated cells later in development" and that "genes normally inclined to remain silent in differentiated cells are retained intact and can be reactivated"[6]. Then, another definition of differentiation is that it is a qualitative change in a cell, relative to another cell, involving a change in the gene activation pattern, i.e., transcription[7]. Within the cell, what steps occur in the differentiation process? As differentiation proceeds, the appearance of each new cell type is linked to the production of unique proteins. These may be structural in nature, such as keratin fibers within the epidermis or collagen that forms outside the cell. Other proteins might be enzymes or diverse groups of enzymes that direct the synthesis of carbohydrates, lipids, or other organic molecules. Protein formation is initiated by the activation and expression of different genes in a particular cell (differential gene expression) that could be triggered by the same genes or might differ from those in another differentiating cell. Not only do special cells such as muscle or blood cells produce proteins unique to their function, but also the kinds of proteins formed in tissues (such as blood) may vary between different phases of development, e.g., embryonic, fetal, or adult. Just which genes are expressed in the production of a specific protein is a consequence of many regulatory events. The DNA of each chromosome is bound with proteins that regulate which genes are available and how specific genes are copied. Adjacent to the gene itself there are two promoter regions located “upstream” from the initial point of gene activation. For example, the exposure of specific gene sequences in the DNA is regulated or may be hidden by histone proteins on the DNA that must be unmasked before a new protein is synthesized. Other processes help control which genes are available for activation. There may also be other regulatory regions on either side of the gene that act as enhancers and suppressors of the activated gene sequences. Enhancers are small lengths of DNA that bind proteins called transcription factors that control genes.

The information for producing a specific protein is coded in the sequence of nucleotide structure exposed in the nucleic acid (DNA) of the cells’ chromosomes. A nucleotide consists of a sugar (deoxyribose in DNA) linked to one of four different bases (organic carbon-nitrogen rings).

The initial event in a given cell is a copy of the DNA of a specific gene or group of activated nuclear genes in the presence of an enzyme. This process is called **transcription** and involves making a copy of a portion of the DNA strand with nucleotides from a second nucleic acid, ribonucleic acid (RNA) using an enzyme (RNA polymerase II). The area bound is called a *promoter*. RNA polymerase often requires various proteins to locate the promoter and these are known as *transcription factors*. The selected DNA portion copied is in the form of a transcript that can be the same or may vary from cell to cell. Even if the copies are identical, two differentiating cells may selectively filter out parts of the transcript so that identical transcripts can produce separate messages in different cells. The entire transcript often contains intervening portions that are not part of the protein coded, but are necessary for processing the RNA within the nucleus.

Ultimately, the RNA may be transported from the nucleus into the cytoplasm where it becomes messenger RNA (mRNA). Once in the cytoplasm, mRNA moves to a protein-producing site of ribosomal RNA (ribosome). It is here where **translation** of the message into a protein is accomplished by assembling amino acids into chains (polypeptides) from a pool of amino acids using transfer RNA, but guided by the template provided by the mRNA. Each amino acid delivered to the protein-forming site is coded by a different triplet of three bases called a codon. These polypeptides are then molded into a three-dimensional protein whose form varies according to the sequence of amino acids. It is apparent that the process of differentiation follows a complicated itinerary both for developing embryonic cells and activated stem cells.

## Differentiation and Restriction of Cell Potential

As differentiation proceeds, two converse events take place. First there is a reduction in the overall potential of the embryonic cells as diversity takes place. The totipotency of the fertilized egg and early cleavage stages is followed by the pluripotency of the cells in the ICM for developing all types of cells, tissues, and organs. As different embryonic systems evolve, such as the nervous system, other cells remain that retain diverse potential for developmental choices within the system. They are multipotent cells and occupy a key position in directing the process of differentiation. These precursor cells have the potential to follow multiple developmental pathways leading to different kinds of cells within a developmental sphere of the embryo.

At the same time, the more specialized a cell becomes, the less is its ability to form other cell types. Not all embryonic cells develop directly into their most specialized state. They usually journey through a separate directed pathway, progressing through a sequence of intermediate stages, each stage becoming more defined, often interacting with other cells, before producing a definitive precursor cell. Some of these may be retained as stem cells. At the same time that their developmental destiny becomes more defined, their ability or potential to pursue alternate pathways is sharply restricted as interaction with cells from an adjacent pathway takes place. An example of this can be seen in the developing lens of the eye. The eye is derived from a part of the neural ectoderm that forms the nervous system. The major part of the eye is later formed from a lateral outgrowth of the brain, the optic vesicle that grows toward the exterior of the head. The lens, however, is derived from the epidermis of the head adjacent to the developing eye. Initially, the epidermis is instructed to form a lens by a signal from the endo-mesoderm layers. Later the presumptive lens epidermis is stimulated by the optic vesicle to form the primordium of the lens (lens vesicle), but develops no further unless it is stimulated by the neural retina to form lens precursor cells that form the lens fibers in the final lens tissue as diagramed below.

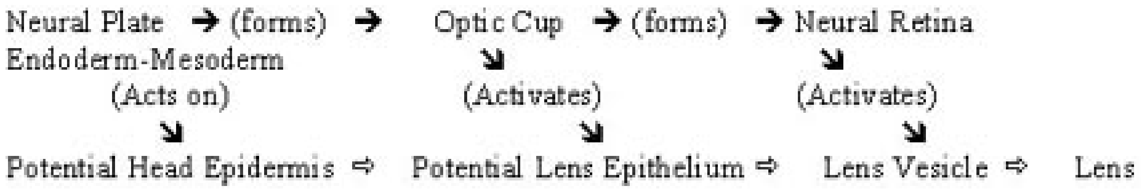

Each definitive tissue may have its own stem cell, for example, the retina that is discussed later.

## *The Potential of Early Embryonic Cells*

The totipotency of the fertilized egg is not necessarily lost at the first cell division.

When an amphibian newt egg is separated with a fine hair loop after fertilization, each separated half of the embryo is capable of forming a complete embryo, provided that each half receives equivalent types of egg cytoplasm[8]. The same is true of other animals where four separate embryos can be produced. The natural separation of early blastomeres is the basis for one method of identical twinning in mammals, each cell being totipotent. Throughout the animal vertebrates, the maximum number of natural identical twins (derived from one egg) is about eight[9]. In humans, the highest number of *identical* twins all derived from early cleavage cells[10] seems to be five (quintuplets), although an unconfirmed report of octuplets has been cited. Other higher multiple births may occur from two or more eggs (fraternal twins), but sometimes they may be in combination with identical twins from a single egg. In identical twinning, each twin is an exact genetic duplicate of the other. In humans, identical twinning may be the result of an asymmetrical division of the blastomeres likely to occur at the three-cell-stage embryos. Rarely, blastomeres may separate again at subsequent divisions to form quadruplets or perhaps quintuplets. The totipotency of individual blastomeres becomes restricted about the eight- to sixteen-cell stage. At the eight-cell stage, two to three blastomeres can be removed without affecting further embryonic development of the remaining cells. The remaining cells are able to accommodate this loss and are reorganized in harmony with each other by a process known as embryonic *regulation*. Regulation is the ability of individual embryonic cells to reverse their destined contribution to development and reorient into a whole embryo. The early totipotent cleavage cells, however, are not considered as stem cells. For example, separation of the two- or four-cell-stage sheep embryos into isolated blastomeres, followed by implantation in foster mothers, produced either identical twins or quadruplets demonstrating their individual totipotency[11]. If left intact, these blastomeres would not form a reserve self-replicating population of cells for use at a later time and by definition they are not stem cells. However, identical twinning in humans generally results from the development of two ICMs within the blastocyst or the subsequent separation of a single ICM. Roughly two-thirds of all identical twins are derived by this method. Each ICM then forms a monozygotic or identical twin. More rarely, identical twinning can also occur at a later stage when dual individuals may form in the late bilaminar blastocyst. As the ICM develops, an embryonic anterior-posterior axis is predicted. Sometimes two embryonic axes appear on separate areas of the embryonic disc of the ICM. The ICM is reorganized into two distinct embryos, each of which would have formed only part of the embryo had they developed as one. If this split occurs after most of the external embryonic membranes have formed, the result may be conjoined identical twins. Nevertheless, all of the identical embryos are derived from the original fertilized egg and have the same genetic makeup.

## *The Potential of the Nuclear Genome*

### Nuclear Transplantation

A fundamental question addressed by early embryologists from the 1880s on was whether the genomic potential of the embryonic nuclei decreases or is lost as cells differentiate into mature somatic cells of the adult. Various early experiments by Jacques Loeb were performed to isolate the nucleus by centrifugation to test the developmental potential of the nucleus in successive cells of the early dividing embryo. A key isolation experiment was conducted by Spemann[8] when he used tiny hair loops to manipulate the eggs of the amphibian newt. At the eight- to sixteen-cell stage, he was able to divert one of the sixteen nuclei into a separate compartment of the egg cytoplasm. The isolated portion of the egg containing a single cleavage nucleus was then detached from the rest of the developing embryo. Both the single nuclear embryo and the original multiple nuclei embryo were able to develop into the larval stage. This indicated that the early cleavage nuclei retained the potency of the original egg nucleus.

The same question of whether the genomic ability of the somatic cell nucleus was equal to the egg nucleus was approached by landmark experiments by Briggs and King[12] on cell nuclear transplants of the leopard frog embryo, *Rana pipiens,* into single egg cells. The basic procedure for these experiments that laid the foundation for nuclear cloning was to isolate separate embryonic cells, remove an individual nucleus from one of them by means of a micropipette, and then transfer the captured nucleus into a recipient egg cell that had its own nucleus previously removed or inactivated (Fig. 2).

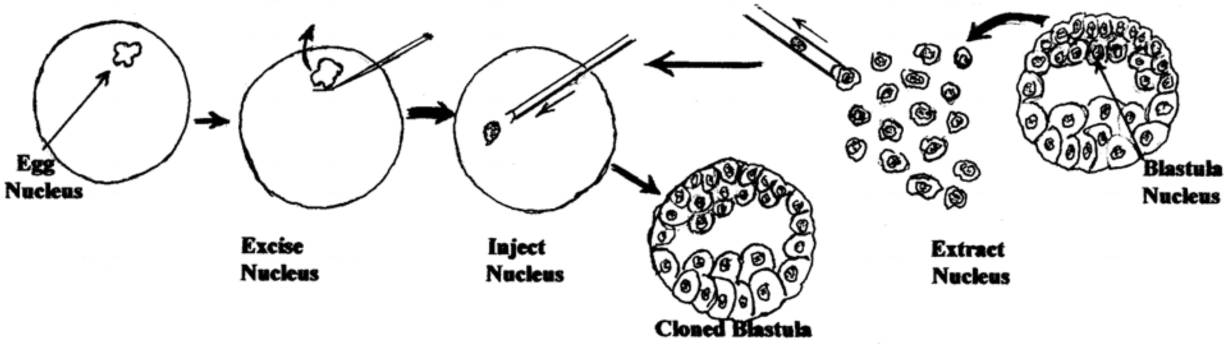

**FIGURE 2**

Single eggs of the frog (oocytes) were activated (normally done by the sperm) by pricking them with a fine glass needle. This triggers movement of the egg nucleus toward the egg surface when the entire nuclear chromosomal apparatus is plucked out, leaving the oocyte without any nucleus (anuclear). The anucleated, activated oocyte now serves as a host for donor nuclei. Next, blastula embryos are taken apart and an individual cell from the blastula stage frog embryo (now consisting of thousands of cells) is taken up by a glass micropipette. The extracted cell is ruptured in the withdrawal process releasing the nucleus. The nucleus with a minute amount of cytoplasm is then injected into an activated oocyte. It was found that up to 80% of the eggs inoculated with blastula nuclei were able to complete their development of embryos into normal tadpoles. This indicated that when the nucleus of a blastula embryo cell was returned to the environment of an egg cell, it would be redirected to act as the original nucleus of the fertilized egg and was fully capable of forming a highly differentiated, functioning tadpole[13].

## Embryonic Nuclear Clones

In succeeding experiments, Briggs and King interrupted development of one of the original nuclear transfer blastula stage embryos and used it as a new donor of nuclei for a second generation of transplants. This first-generation blastula, now containing multiple duplicates of the original donor nucleus, was disassociated into individual cells and nuclei from the cells were injected into new anuclear oocytes. Each of these recipients developed into normal frog embryos. This process of serial transplantation is known as *nuclear* cloning. These experiments established the pluiripotency of blastula cell nuclei and the methodology became the basis for other studies including cloning in other animals. In contemporary usage, each member of a generation of embryos is considered as a clone.

Similar experiments of Gurdon and colleagues[14] were conducted on eggs of the South African clawed frog, *Xenopus,* using slightly different techniques. The host egg oocyte was irradiated with ultraviolet to destroy the maternal nucleus that stayed behind and nuclear markers were used to distinguish the host and donor nuclei. They also found that blastula nuclei transplanted into oocytes could produce swimming larvae and in one case, a larva developed into a mature adult frog. Later separate experiments between species using genetic markers conclusively demonstrated the totipotency of the blastula nuclei when reproducing frog adults were produced in both species[15,16]

Significantly, nuclei transplanted[17] from partially differentiated cells of the tadpole intestine into anucleated eggs produced a similar event. While most embryos became arrested, one of the eggs with a transplanted nucleus appeared to be totipotent when a larva metamorphosed into a fertile adult frog. However, it was not shown unequivocally that the introduced nuclei came from differentiated cells.

As nuclei were selected as donors from later and later embryos, the success of development began to decline, both in the leopard frog and clawed frog experiments. However, attempts to transplant nuclei from differentiated adult frog cells provided mixed results. The nuclei of terminally differentiated adult frog red blood cells were transplanted into activated, anucleated oocytes[18]. The donor nucleus contained a nuclear (triploid) marker. In about 8% of the transplants, the blood cell nuclei directed development of the embryos into feeding tadpoles approaching metamorphosis when development ceased. These and other experiments indicated that nuclei from differentiated larval and adult frog cells are pluripotent, but no adult amphibian cell nucleus has been shown to be to be totipotent[13].

The quest for producing clones was extended to mammals. In most cases, nuclei were obtained from early preimplantation embryos. Early reports with cells from the ICM embryos of mice were not verifiable. The experiments were extended to domestic animals where the nuclear transfer technique used cell fusion rather than nuclear injection by microinjection. In this procedure, nuclei are allowed to enter the anucleated cell by inducing the nuclear donor cell membrane and the recipient host oocyte membrane to fuse by an electrical discharge, creating a new cell with a single nucleus. Using this technique, Willadsen[11] was able to produce adult lambs when he enucleated oocytes and then fused them to single embryonic cells from eight- to sixteen-cell-stage lamb embryos. After implantation into surrogate mothers, they gave birth to cloned lambs. The same success was soon obtained with embryos from assorted domestic and other animals[19].

Experiments with primate embryos[15] were also successful. Nuclei from an embryo of the rhesus monkey fertilized in a culture dish (*in vitro*) were obtained after separation of early cleavage blastomeres. A genetically different adult provided oocytes that were enucleated with a micropipette. An eight-cell blastomere with a donor nucleus was then placed in contact with the enucleated oocyte and the two cells fused with an electrical current (electroporation) that perforates the cell and allows the donor cell nucleus to enter the oocyte. Following culture *in vitro*, the embryos were transferred into foster mothers. This resulted in the birth of two adult rhesus monkeys derived from the transfer of embryonic nuclei.

The first adult somatic cell nuclear transfer in higher animals was produced by Wilmut et al.[20] when they selected a nucleus from a mammary gland cell line obtained from a sheep ewe. Using a donor oocyte from a blackface ewe that had been enucleated with a micropipette, the mammary gland nucleus was introduced into the oocyte by electrofusion. Following several days in culture, the embryo was transferred to a surrogate ewe that gave birth to Dolly. She in turn has given birth to several offspring. This experiment clearly established totipotency of the reconstructed oocyte and that differentiated adult nuclei were fully capable of directing the entire development of the embryo into a reproductive adult. The degree of success, however, is very low and may be due to inactivity of key embryonic genes[21]. Subsequently, somatic cell nuclei from several different animal cells have been used to produce new embryos and adults in a variety of animals, cattle, pigs, and mice[13].

The procedure of nuclear cloning, so important to applications of animal reproduction, has had a great impact in the process of animal cloning, but also has a bearing on the production of stem cells and related areas.

## Embryonic Chimeras

Paradoxically, the early mammalian embryo stages capable of forming multiple embryos are so versatile that they can also fuse and assume one identity, an inverse of twinning. This is the result of embryonic regulation (described above). Milestone experiments on the regulatory potential of early embryonic stages of the mouse were accomplished by the aggregation of blastomeres from two different mouse strains[22,23]. Two separate early cleavage stages, from eight to sixteen cells, of mouse morulae embryos were recovered and their protective gel-like layer (zona pellucida) removed. When the two embryonic cell masses were pushed together, they fused and developed into a single integrated blastocyst. The blastocyst was then implanted into the uterus of a foster parent and allowed to develop (Fig. 3). When embryonic cells from genetically different mice are used, identified by several characteristics including coat color, they form a mosaic blastocyst (ICM + trophoblast) from a complete amalgamation of the cells from two different mice. The resulting embryo is a chimera derived from each of the two genetic strains joined into one heterogenous embryo, but normal in all respects. The ensuing adult mouse often has a striped coat color derived from each of the fused embryos. Even three (six parental) embryos can be fused in this manner[24]. Thus, each of the embryos became integrated into one embryo capable of forming all of the cells and tissues in the adult. This integration reaffirmed that development in these early cells was not rigidly established (determined) and that the blastomeres could regulate or reformat their developmental destiny. In a sense, the outcome reaffirmed that early blastomeres still possess a vast developmental potential. Other experiments confirmed this. Blastomeres isolated at the two-, four-, or eight-cell stage appear to have the potential to form essential products of the entire embryo, i.e., the outer external embryonic membrane layer, the trophoblast, and the ICM[25]. In the early blastocyst embryo, certain cells remain as pluripotent cells, lacking the totipotency to form an entire embryo and its external membranes, but capable of forming all of the specialized cells, tissues, and organs of the entire embryo. They are cells that constitute the ICM and include those that will become the future germ cells of the organism.

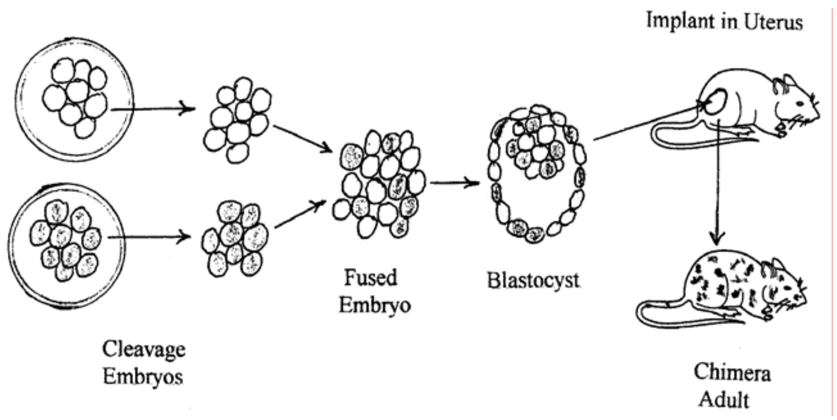

**FIGURE 3**

## Pluripotency, Embryonic Tumors, and Stem Cells

During normal embryonic development, certain cells are set aside to become the future reproductive cells of the adult. These are primordial germ cells (PGCs), precursors to the cells that form into eggs or sperm. They will migrate into future regions of the reproductive organs (genital ridges ) that form the testis or ovary (gonads). Sometimes, the PGCs are the source of developmental abnormalities (teratomas) that occur spontaneously in both mice and humans. If the genital ridges with PGCs of a teratoma-producing mouse strain are transplanted into the testes of adult mice, greater than 75% will form teratomas in their testes, indicating they are derived from PGCs[26]. These abnormal growths consist of a grossly disorganized mass of differentiated tissues such as muscle, bone, cartilage, hair, and nerves. Often such growths occur in a complex with an undifferentiated rapidly growing type of tumor (carcinoma) forming a tissue mass called a teratocarcinoma. The carcinoma cells resemble embryonic cells since they have specific cell surface markers that are embryonic and seldom found in the adult. These tissue complexes are particularly unusual since they express both highly differentiated cells and tissues, but also possess pluripotent undifferentiated carcinoma stem cells that are indeed versatile. This was seen when injection of the embryonal carcinoma cells into the body cavity (peritoneum) of adult mice produced groups of membrane-covered spheres called embryoid bodies that contained clusters of normal cells resembling early embryonic stages of differentiation. The undifferentiated tumor cells of the teratocarcinoma were also isolated by Mintz and Illmensee[27] and placed into culture outside of the body (*in vitro*). Several cell lines of carcinoma cells (EC) were established as pluripotent stem cell lines that were capable of differentiating into normal cells.

Some of the cultured stem cells were injected into a normal blastocyst where they became incorporated into the ICM. The blastocyst was implanted into the uterus of a foster mother (Fig. 4). The embryo produced a mouse that was a chimera of the abnormal carcinoma cells and of the normal embryo cells confirming the pluripotency of the former cells and their ability to undergo regulation[27]. When some of the EC cells became incorporated into germ cells, such mice could be crossed with normal mice and their progeny had mixed heterogenous chromosomes derived from the abnormal cells and the normal embryo. Further crosses between these heterogenous mice from tumor/normal cells produced diverse genetic combinations. Some of these mice possessed homozygous genes derived from the tumor stem cells and predicted a further use of stem cells as a vehicle for gene transfer. However, as a source of normal stem cells, they possessed various deficiencies[28] such as abnormal chromosomes.

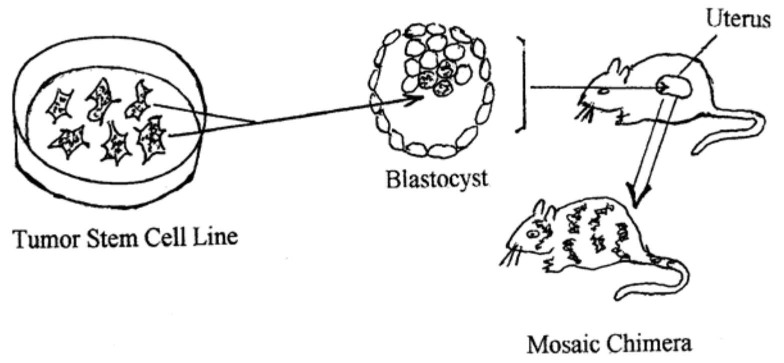

**FIGURE 4**

## Embryonic Stem Cells

Since the cells of the mammalian ICM of the blastocyst appeared to be pliable and not determined to become any specific cell or tissue, attempts were made to recover normal cells from the ICM in order to test the potency of the individual cells comprising the ICM. Two parallel research efforts resulted in isolation of the

ICMs from mouse embryos. The aim was to separate the ICM from the premembrane trophoblast layer and grow the ICM-derived cells in culture so that presumably pluripotent stem cells could be recovered. To accomplish this, it was necessary to stimulate their cell division, but prevent differentiation of the cells from the ICM. The culture and recovery of embryonic stem (ES) cells from the ICM of normal embryos involved one of two isolation techniques. In one case, the premembrane layer of the isolated blastocyst was treated with antibodies to destroy and remove it. This exposed the ICM that was then placed into tissue culture on feeder cell layers containing conditioned culture medium with a factor from the tumor (carcinoma) cell line (Fig. 5). This produced embryonic cell colonies.

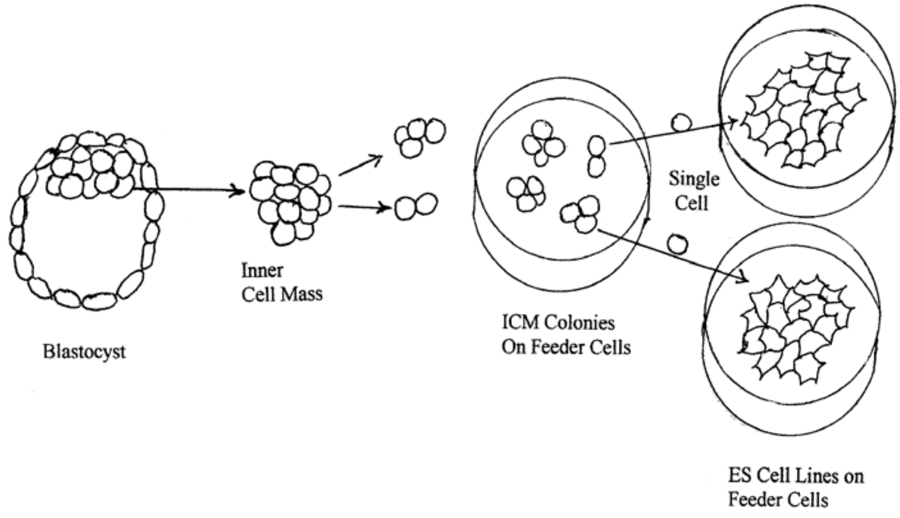

**FIGURE 5**

Selection of single cells with undifferentiated characteristics from these colonies produced a line of pluripotential ES cells[29]. The second procedure began with the delayed recovery of blastocysts from a hormone-treated adult that prevented implantation and stimulated cell division, but prevented differentiation. Subsequently, the blastocyst was placed into culture allowing the premembrane layer to proliferate and exposing the ICM. The ICM was "picked off" and then cultivated on a special feeder cell layer of inactive embryonic mouse tissue supportive cells (fibroblasts) that had been treated with radiation that allows them to metabolize, but prevents their proliferation.. An inhibitory factor was used to prevent premature differentiation of the ES cells. In this way, a permanent cell line of ES cells (EK) was produced[30]. More recently, the technique has been refined so that blastocysts are placed in culture directly on feeder cells where the trophoblast layer proliferates, spreads out, and exposes the ICM[28]. The ICM is recovered and broken into clumps and cultured again on feeder cell lines. These feeder cells secrete an inhibitor that prevents differentiation, but allows cell proliferation of the trophoblast cells. In practice, a tumor-inhibitory factor (leukemia-inhibitory factor) is added to the culture medium. Small colonies of cells are produced that consist of either differentiated cells or undifferentiated colonies. The latter are selected and regrown to produce lines of pluripotent stem cells. A key advance in human stem cell research came when ES cell lines were established from cells obtained from unused human embryos after *in vitro* fertilization. These stored frozen embryo cells retained in fertility clinics were released by the donor individuals for research. Using the same isolation technique developed to produce mouse ES cells, the early preimplantation embryos (four- to eight-cell stages) were grown to the blastocyst stage. The ICM cells of the blastocysts were isolated and grown on irradiated mouse feeder fibroblast cells. Nondifferentiated cell colonies were selected to yield five continuously growing stem cell lines[31]. These "immortal" cell lines continued to grow without differentiation for 5 months and remained undifferentiated. This was due to the continuous production of a chromosome appendage (telomere) that extends the lifespan of the cells. The lines produce telomerase that perpetuates their culture indefinitely. Furthermore, they maintained a normal chromosome complement (karyotype). When cultured without the feeder lines, the cells would differentiate new cells spontaneously and exhibit proteins and other surface molecules characteristic of nonhuman, primate precursor cells and preimplantation trophoblast membrane cells that produce a pregnancy-related hormone (chorionic gonadotropin). However, when the cell lines were injected into the rear leg muscles of mice lacking an immune response (immunodeficient), they formed highly differentiated abnormal teratomas. Significantly, these highly differentiated clumps of human tissue from the human embryo–derived cell lines produced tissues characteristic of precursor cells derived from each of the three embryonic lines, i.e., gut epithelium, cartilage, bone and muscle, neural tissue, and body epithelium. While individual stem cells per se were not isolated, these experiments indicate the great potential of human ES (hES) cell lines for studying human normal and abnormal development and gene function *in vitro*[32]. In a broader sense, the greater potential of these lines is for the production of lineage-restricted stem cells for heart muscle, nerve cell precursors, pancreas, etc. and their subsequent use in transplantation therapy. More recently, newer techniques have evolved for isolating hES cells that eliminate the need for mouse feeder cell lines.

## Modifying Stem Cells: Transgenic Chimeras

The formation of mosaic embryos through the fusion of disparate ICM cells is also the basis for creating another type of a chimera where ES cells can be altered with foreign genes through a process known as *transfection*. This is a technique where gene fragments from one species are incorporated into the chromosomes of another species, resulting in a transgenic organism. The EC stem cell procedure involved in the production of chimeric mice became the basis for manipulating the stem cells by adding genes from one mouse strain into another genetic mouse line that resulted in a transgenic mouse. First, normal ES cells from the ICM of a blastocyst stage of mouse strain A were isolated and placed into culture. These cultured ES cells are then mixed with multiple copies of specific cloned genes often by the use of cell-penetrating vectors such as retroviruses (RNA containing tumor viruses). The desired gene is first incorporated into the viral genome. Once inside of a cell, they produce a DNA copy of themselves. Retroviruses can insert their own genome (but inactivated since they lack the genes for forming a virus) into the chromosome of a cell and thus serve as a gene carrier in the altered cells without destroying them. Another procedure utilizes an electrical stimulus to open the cell (electroporation) in order to introduce the cloned gene. The altered transgenic stem cells are then cultivated and the ES cells that contain the incorporated transgene are selected and injected into the ICM of a second mouse strain B (Fig. 6).

The blastocyst with cells containing the integrated genes is then implanted into the uterus of a foster parent from strain B, producing a chimeric mouse. If the integrated cells get into the germ cell line, then some of their progeny will carry one copy of the inserted gene. When the chimeric mice that harbor the transgene are mated with a normal strain B mouse, it is possible to produce mice with mixed copies (heterozygote) of the gene found in the normal/chimeric mice. If two heterozygote mice from this cross are mated, a portion of the progeny will carry two like copies (homozygote) of the integrated gene in all of its cells[33]. These latter animals are selected for study of the gene activity. The transgenic technique has also led to a variation of the stem cell procedure where specific genes could be targeted. Instead of adding genes, specific genes are "knocked out" and replaced with a mutant gene.

In this case, a gene is cut by an enzyme and a portion or the entire gene is replaced with a mutant form. Next, the mutant gene is introduced into a stem cell using electroporation. A few of the cells will incorporate the mutant gene naturally. The individuals that acquire the spliced DNA of the mutant gene are called "knock out" transgenic mice. This procedure has become a powerful method for the analysis of the function of targeted genes during embryonic development and is another way that stem cells can be tailored for therapeutic treatments.

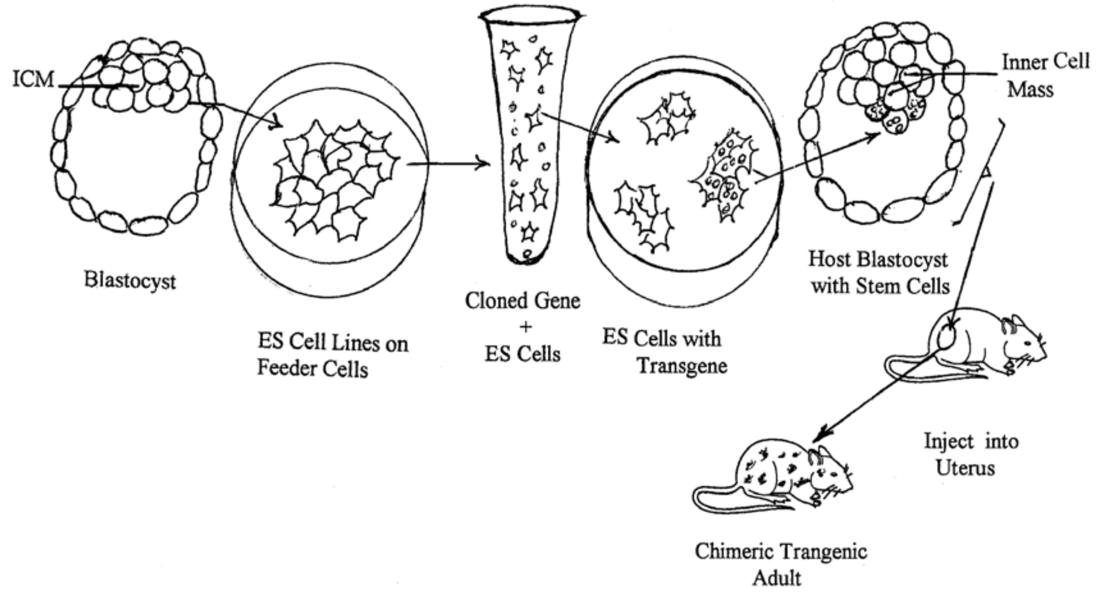

**FIGURE 6**

It should be pointed out that the production of transgenic ***cloned*** animals, constituting a major advance in mammalian cloning and gene targeting, does not necessarily invoke the production of stem cells. Rather it involves a combination of cloning the oocytes of one species with nuclei from donor cells of a different species whose genome has been targeted with a specific gene. For example, transgenic clones were produced by Schnieke and coworkers (illustrated in [13] ) when fetal lamb cells were transfected with a human gene while in tissue culture. The transgenic nucleus was then added to an enucleated sheep oocyte that developed into an embryo. Subsequently, it was implanted into a female adult sheep. The surrogate ewe gave birth to a lamb having a human gene for a blood-clotting factor in her milk.

## Cloning of Stem Cells

A link between the techniques of nuclear cloning of mice and the production of ES cells was demonstrated[34] when cloned mice were obtained from pluripotent ES cells. A previously established ES cell line following extended cultivation was used as the source of nuclei. The nuclei were microinjected into 1,765 previously enucleated mouse oocytes. After reconstruction of the oocytes and their activation, they were cultured up to the morula/blastocyst stages that were implanted into surrogate mothers. From these, twenty-six pups were born, thirteen developed into adults, and one became a reproductive adult that produced several litters. These experiments showed that the ES cell nuclei were totipotent since full embryonic development followed their transfer into oocytes. However, the significance of having ES cell lines, either ES or EG that may be directed into specific cell lineages, is that they could provide an avenue for the production in a single generation clones of ES cells each with an identical nucleus carrying targeted genes. This procedure may become extremely valuable for genetic manipulation through transgenesis for the correction of gene defects, diseases, etc.

## Stem Cell Profile

Not all embryonic cells continue development into fully differentiated cells in the embryo and in the adult some residual cells form a reserve for the replacement of rapidly growing tissues. These are stem cells. The inherent characteristic of a stem cell is that it is able to divide into two cell types, one identical to itself and thus self-perpetuating, and a second new cell that begins to differentiate, usually specific to the tissue in which it is found. The mechanism, observed by early embryologists in certain germ cells, is called asymmetric division. Thus, reserve stem cells can both duplicate themselves to maintain a continuous stem cell population and also have the ability to produce progenitor cells that are programmed toward a specific differentiated fate. Once they are committed to a specific pathway, they are now progenitor cells, but they may still have the capacity for limited reproduction (Fig. 7). At any given situation, some or all of these cells cease dividing and proceed by stepwise maturation to the differentiated state.

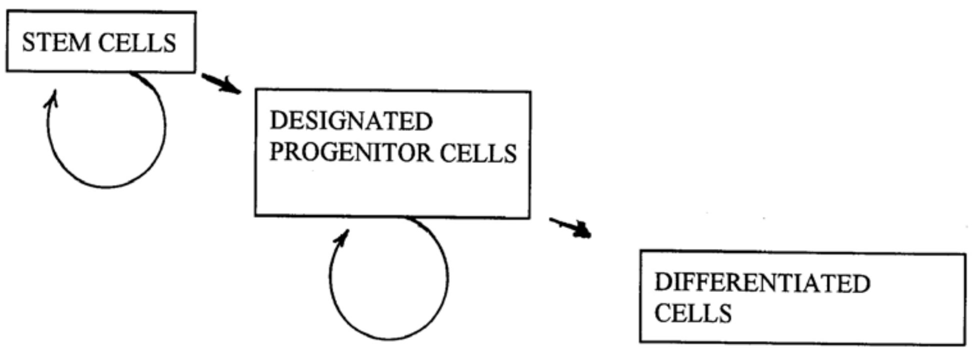

**FIGURE 7**

Since stem cells are maintained through self-renewal and yet are capable of differentiating into precursor and progenitor cells, some genic regulation must be involved. In the fruit fly, *Drosophila*, the PGCs develop into germline stem cells (GSCs) that are maintained through self-renewal. From this stem cell pool, some cells differentiate into specialized cytoblasts in the larva or pupa. Ultimately they enter an egg development process (oogenesis) that produces adult egg cells. A study on the ovary[35] has implicated a NANOS family of genic translation repressors. They found that the NANOS repressors are necessary for the self-renewal of GSCs and that regulation of gene translation prevents stem cell entry into the differentiation pathway. If similar events take place in mammals they may lead to stem cell activation. .

Loeffler and Potten[7] proposed that activated stem cells (compared to potential stem cells) of a particular tissue have the following characteristics:

- Undifferentiated relative to their (ultimate) cell offspring (progeny)
- Capable of proliferation and able to maintain their population
- Able to produce differentiated, functional cell progeny
- Able to regenerate a group of differentiated functional cell progeny after an injury

Somatic stem cells in the embryo are such populations of cells. They generally function during a specific phase in embryonic or fetal development. They do not necessarily persist into the adult, but analogous stem cells may develop and parallel their function in the adult. Thus, not all adult stem cells are leftover embryonic or fetal cells. In mammals, similar adult stem cells self-renew and form progenitor cells. In most tissues of the adult, stem cells may constitute only one cell in many thousand cells, may divide slowly or not at all, and are only isolated by recognizing specific molecules that are found on their cell surface. Since they may resemble cells from the mature tissue, this may involve a complicated cell-harvesting process.

There are at least two hypotheses on the developmental origin of stem cells. One suggests that there are multipotent residual stem cells cached away in all tissues that can be activated as needed and directed along a particular cell or tissue lineage. This would favor an embryonic or fetal origin of multipotent stem cells that persist into the adult. A second view is that there are separate, more restricted stem cells in the progenitor state specific to a particular cell or tissue type such as muscle. These cells might be found resident in the same muscle tissue or widely distributed on call as needed. Conceivably each type of stem cells coexist.

These concepts are relevant since they relate to evidence that stem cells from various tissues of the adult, presumably committed to being a reservoir for a family of related cells such as those found in the bone marrow (hematopoietic stem cells), can be changed under appropriate environmental signals and reprogrammed to regenerate diverse unrelated cells such as heart muscle cells[36]. Some would argue that rather than reprogramming stem cells to regenerate a particular tissue, there may be widespread distribution of cell-specific stem cells such as those for muscle located in distant compartments that could be recruited as such when needed.

Embryonic stem cells derived from the ICM or PGCs differ in that they have the ability to form all cell types found in the embryo or future adult and thus are pluripotent. They do not have the potential to form a complete organism, but may form all cell types and tissues found in the adult. Once stem cell lines are derived from them, they proliferate in the undifferentiated state (self-renewal), but retain the ability to differentiate when culture conditions are modified. A variety of stimuli and signaling molecules are used to direct them into specific cell lineages.

Other stem cells, associated with a particular system such as the blood-forming system in the bone marrow of the embryo or adult, have a more limited developmental lineage and since they form only certain cells found in the blood such as red blood cells and various types of white blood cells, they are defined as being multipotent. Unfortunately, contemporary investigators of stem cells have sometimes used the terms pluripotent, multipotent, and even totipotent interchangeably although the distinction between pluripotent and multipotent is arguably semantic. However, these distinctions are generally accepted[37]. The fertilized egg is totipotent since it can generate the entire embryo and its membranes, but it is not considered as a stem cell. Cells from the ICM or from the PGCs are pluripotent stem cells since they can form all of the cells and tissues of the entire embryo (but they do not normally form extraembryonic membranes, nor do they form the entire embryo). Stem cells found in a more specialized tissue may be multipotent and self-renewing, but they have a more limited potential. Finally, many external events such as growth factors, the removal of inhibitors, and the effect of gene-controlled cell transcription factors may promote entry of multipotent stem cells into a specific differentiation pathway[38]. As progenitor cells, they are then committed to a specific pathway with limited self-renewal and are subject to a variety of extracellular environmental signals including growth factors, cell regulators (cytokines), and hormones. Further regulation of stem cells during differentiation can be produced by cell-to-cell interactions, plus signal transduction pathways that regulate transcription factors and extracellular proteins (extracellular matrix) found in the outer domain between cells.

## The Role of Growth Factors

As embryonic cells divide and differentiate, they are stimulated to grow by small proteins called growth factors. These are molecules, much like hormones, that when bound by a cell can regulate cell division or differentiation. Different kinds of cells require one or more growth factors. They may respond to their own factor or to other growth factors by the presence of specific receptors located at the cell surface (plasma membrane). Growth factor receptors on the plasma membrane of the embryonic cell may bind to specific growth factor proteins (ligands). The majority of these receptors belong to a specific enzyme family (protein kinases). A large number of growth factors exist, their names often describing their first known function, but their range of activity is often more extensive. In the developing embryo or fetus, growth factors are involved in the genesis of a variety of normal tissues or organs ranging from germ cell proliferation, muscle differentiation, regulation of how the brain is organized, both normal and abnormal skeletal formations, kidney formation, blood vessel expansion, limb development, and even limb regeneration[33].

The developing nervous system depends on a primary growth factor. More than 50 years ago, an embryologist, Victor Hamburger, and a neurobiologist, Levi-Montalchini, noted that when a limb bud was removed from a developing chick embryo, the associated sensory nerves and clusters of nerve cell bodies (ganglia) would undergo atrophy. However, the addition of limb buds to a normal embryo would increase mitotic activity and enhance differentiation of the sensory ganglia. They postulated that this increased activity was caused by the diffusion of some stimulating trophic agent. Later studies were extended to mice when it was found that a mouse tumor (sarcoma) had the same effect on the chick sensory ganglia. The factor that enhanced growth of sensory nerves and sympathetic ganglia was defined and isolated by Montalchini and Cohen (for which they were awarded the Nobel prize) as a nerve growth factor (NGF)[33]. This polypeptide molecule stimulated cell division, cell growth, and enhanced differentiation and a primary source in the embryo was the salivary gland. Another growth factor recovered from the salivary gland was an epidermal growth factor (EGF) that also stimulates cell division and growth of epidermal cells as well as keratin formation[39]. Some growth factors have restricted cell targets while others having expanded targets and functions[40]. The EGF triggers epidermal cells to grow, but also has an effect on other cell types. It is known that the basic fibroblast growth factor (bFGF) consists of a family of separate gene-controlled factors, each regulating normal developmental phases such as blood vessel proliferation or the fiber-like extension of nerves (axons). Certain mutations may produce abnormalities in bone formation or brain development by altering either the growth factor or its receptor[33]. Another group of hormone-like proteins are cytokines (cell growth regulators) such as interleukins, originally discovered in cells involved in the immune response mediated by specialized blood cells, the lymphocytes, and macrophages. A family of interleukins (IL-1, 2, etc.) stimulates cell proliferation, differentiation, and other functions of stem cells. Even more startling is that in certain circumstances, these growth factors may have an inhibitory effect. A cytokine originally found in transformed rodent cells, the transforming growth factor (TGF beta), also detected in normal cells, was found to be both inhibitory to epithelial cells including mammary duct cells *in vitro,* but also stimulated other cells to grow[41]. Other factors are only inhibitory in nature so that, taken collectively, growth factors and inhibitors are often referred to as growth regulators. Growth factors often perform important links in the process of cell differentiation. Such is the case of the fibroblast growth factor (FGF). Neural progenitor cells of certain sympathetic nerves require the presence of the NGF. However, they cannot respond until they become receptive (competent) to NGF through the presence of specific receptors for NGF on their surface that they lack in the progenitor cell state. The action of FGF on the neural progenitor cells causes the formation of NGF receptors on the cell surface. This in turn allows the binding of NGF to the undifferentiated neural cell leading to its maturation into a mature sympathetic neuron[33]. Growth factors are not confined to their primary function as the NGF is also responsible for differentiation of the fundamental unit (nephron) of the developing definitive kidney. Growth factors are also operative on cells grown in culture and as such, influence and can control and expand stem cell potential.

## Stem Cells Active in Normal Embryonic Development

### *Neural Crest Cells*

One of the most complex, intriguing, and extensive sequence of embryonic pathways leading to specialized cells and tissues is found in the neural crest cells of the early developing embryo[42]. While their potential to form any cell of the embryo is partially restricted, they may still follow a broad spectrum of developmental fates. Prior to the formation of the nervous system, the embryo is covered with a layer of cells, the ectoderm, that is destined to form the surface layer of the body and a separate demarcated area that will ultimately form the entire primordium for the nervous system, the brain, the spinal cord, and attendant nerves. Oriented parallel to the longitudinal body axis on the future back (dorsal) side, this primordium of the nervous system begins a lengthwise inward folding of the surface ectoderm cells that will generate a long groove that gradually folds into a tubular structure, the neural tube. The neural tube pinches off and sinks just beneath the surface of the embryo.

Some of the cells within and adjacent to the neural tube will cease cell division and form specialized embryonic cells called neuroblasts. These are embryonic precursor cells that can give rise to neurons that either become sensory nerve cells (transmitters of sensory-derived information) or motor nerve cells (transmitters of some action). Extensions of the differentiating neuroblast are nerve fibers that convey impulses into or out of the surface of the embryo. Ultimately, the neural tube will form the entire brain and the continuing attached spinal cord. Other cells to form in the neural tube are neuroglia cells, embryonic precursors to cells that constitute a supporting network (glial cells) and make up the bulk of the cells in the brain. Their function can be protective, but they also provide maintenance to neurons by removing waste and providing nutrition as well as aiding in transmission of nerve impulses along the neuronal axon; they also direct development and transmission of nerve impulses across connections of neurons (synapses) to other nerves, sensory organs, muscles, etc. In addition, a functionally separate group of neurons, the autonomic nerves located outside of the central nervous system, connect with the nerves of the brain and spinal cord. These nerves are responsible for involuntary nerve-controlled activities of the internal organs (viscera) and influence such activities as breathing, digestion, etc. A subdivision, the sympathetic nerves, is often associated and is concerned with emergency activities.

As the neural tube closes, a group of cells separate peripherally from the neural tube in the head and mid-body or trunk regions (see Figs.8 and 9). These are the neural crest cells[43].

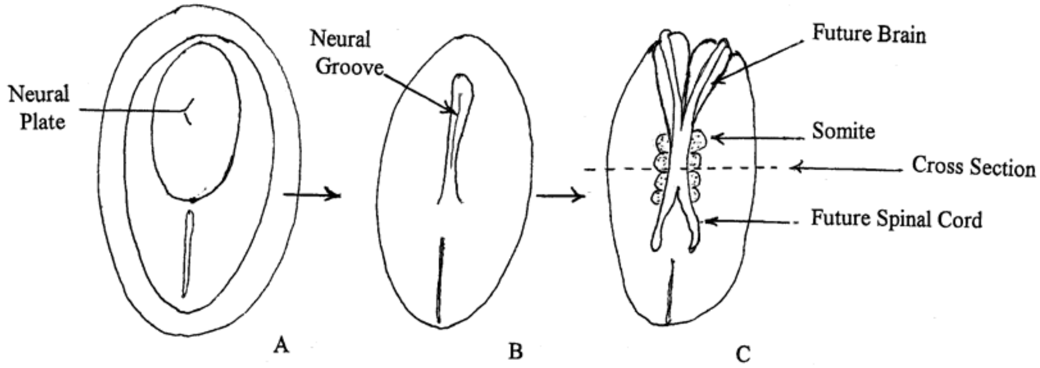

**FIGURE 8.** Formation of a nervous system.

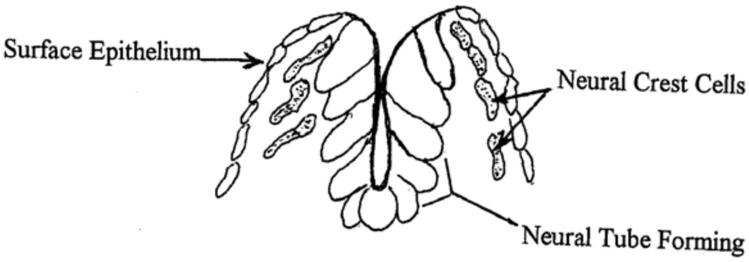

**FIGURE 9**. Neural crest cells (cross-section of “C” in Figure 8).

The neural crest cells are pluripotent ES cells that migrate to other regions of the embryo where they are stimulated to form a variety of cells, depending on signals they receive at their destination site. Once arrived at a site, they may form nerves and groups of nerve cell bodies (ganglia) that receive or send impulses via nerve fibers. In particular, they will form ganglia of the sensory nervous system in the brain and spinal cord plus many ganglia of the peripheral autonomic nervous system. Some of the migrating neural crest cells will generate pigment cells when they arrive in the skin; others are destined to form certain cartilage, facial skeleton, and muscle cells of the future head region. Another group will stream down into the embryo to form the parts of the developing thyroid, parathyroid, and adrenal glands that become hormone-producing cells. A signature of neural crest cells is their potency to form alternate cell types within the developing embryo, given the opportunity. For example, the ultimate fate of neural crest cells migrating from the trunk region is first established by the migratory pathway guided by a specific adhesion molecules and molecular signals. One of these signals is a stem cell factor, a protein produced by the cells along the migration pathway that is bound to a receptor site on the cell membrane. Those cells that migrate along a superficial pathway beneath the ectoderm will usually form pigment cells.

The final differentiation of neural crest cells into either sympathetic neurons or hormone precursors (chromaffin cells) of the adrenal gland involves a number of external stimuli. The latter pathway invokes steroid molecules (corticoids) that first inhibit neuronal development and then stimulate specific enzymes leading to the development of chromaffin cells. The pathway leading to neuron differentiation requires the presence first of a bFGF and then NGF for their differentiation into sympathetic neurons[33].

Multipotency of the neural crest cells exists prior to cell migration. Normally, the neural crest cells adjacent to the anterior brain (forebrain) do not form neurons, but contribute to cartilage around the developing eye. If part of the neural crest adjacent to the developing chick forebrain is transplanted into the trunk regions of quail embryos, the chick embryo cells distinguishable from quail cells develop into sensory and autonomic nerve cells that are part of their pluripotential, but not their normal, destiny[42]. Lineage studies within the living embryo (*in vivo*) have demonstrated the intrinsic potential of neural crest cells and their ability to form diverse cell types. To establish that single neural crest cells are multipotent before migration, individual neural crest cells were injected with fluorescent molecules before they migrated from the neural tube area. The progenies of these cells, readily identified by their fluorescence, were later found in sensory neurons, supporting nerve cells, pigment cells, and endocrine cells of the adrenal gland[44].

Neural crest cells, from their role in a dynamically changing embryo, might be assumed to be transitory, only serving a role in a stream of embryonic development and having a limited differentiation capacity. However, given the opportunity, their ability to continue self-replication and to express their potency was aptly demonstrated after recovery of neural crest cells from mouse embryos and their growth outside of the embryo in tissue culture. Isolation of individual cells and proliferation of their progeny produced clusters of identical cells that were self-renewing. Such cultures were able to differentiate into neurons and nerve-supporting glial cells that indicated the existence of a true stem cell[45]. They were also able to form precursor smooth muscle cells (myoblasts).

The action of growth factors on stem cells is often affected by other gene-controlled signaling pathways known as signal transduction pathways. They trigger the movement of information from the cell membrane to the nucleus in response to extracellular signals and control gene expression and cell proliferation. One of these, the Wnt pathway, consists of a large family of secreted glycoproteins that bind to cell surface receptors and induce an alteration in the expression of specific genes. Thence, the Wnt protein may induce cell proliferation or differentiation during embryonic development by initiating a signaling cascade within the cell that can modify growth factors. Both the induction of neural crest stem cells from the neural epithelium and their growth are controlled by the Wnt genes in mice and the frog *Xenopus* that encode for the secretory signals[47,48]. After signaling by Wnt, a series of proteins inside the cell ultimately interact with a protein beta-catenin. This protein enters the nucleus where it forms a complex with a major transcription factor that induces proliferation of the neural crest cells.

The same protein involved in the induction of neural crest cells also controls the formation of melanocytes when it was shown that removal of the Wnt/beta-catenin gene caused a loss of melanocytes and sensory neural cells of the dorsal root ganglia[48]. It now appears that the Wnt/beta-catenin signal is able to direct neural crest stem cells specifically into sensory neural cells in mouse embryos[49]. Mutant mice were selected that lacked the normal neural crest–derived cells in the forebrain region. Induction of sensory neurons in this region (normally lacking neural crest derivatives) was produced on continuous activation of beta-catenin. In addition to triggering neural crest cell formation, they found that another function of Wnt/beta-catenin is instructing the emigrating crest stem cells to form sensory neurons.

There are several stem cell systems active in the embryo besides neural crest cells. These include the precursors to the germ cells (PGCs), the blood cell (hematopoietic) stem cell system, as well as epidermal stem cells and recycling cells of the small intestine epithelium, all similar systems that are also found in regenerative stem cell systems of the adult.

## *Primordial Germ Cells*

Early in development, depending on the species, special precursor cells to the future germ cells, the PGCs, are established in a germ layer located in an external embryonic membrane (extraembryonic endoderm). These cells differ from all other somatic cells by possessing a special cytoplasm (germplasm) resulting from the production of a unique cytoplasmic factor, first expressed in the ICM of the blastocyst in mammals. PGCs have the potential to produce both future germ cells and all types of somatic cells and thus are pluripotent cells. In the early developing embryo, the PGCs migrate into the developing testis or ovary (gonad). After their arrival in the gonad, the PGCs divide and then transform themselves into a population of gonial cells. The female has a fixed supply of these precursor cells that are nonproliferating. In the late developing male, residual stem cells form and are retained in the adult male testis as progenitor cells to provide a continuous supply of mature sperm cells that are generated cyclically throughout the reproductive life of the adult. Transition into a (spermatogonial) cell is usually completed about the time of birth. When the adult male reaches reproductive age, the spermatogonial cells resume division as a population of gonial stem cells that are self-proliferating. However, some of these cells may change into reserve progenitor cells. This population of progenitor gonial cells (Type A) are relatively nonproliferating. Predictably, part of the Type A cells cyclically embark on a sequence of differentiation leading to the transition into cells known as Type B gonial cells. Continued productions of Type B cells produce sperm precursor cells (spermatocytes) that eventually differentiate into mature spermatozoa.

### Stem Cells from Primordial Germ Cells

In mammals, PGCs can first be identified in the future external embryonic membrane area. These cells are recognized by a stain for a specific enzyme (alkaline phosphatase) and the use of antibodies to cell surface molecules[33]. PGCs were isolated directly from 7-day-old mutant embryos that lacked the stem growth factor and were cultured on a mouse embryo feeder cell layer (STO) in the presence of the leukemia inhibitory factor (LIF), a "steel" factor, and bFGF[50]. The PGCs proliferated and produced large, long-term colonies of cells that shared characteristics of earlier isolations of ES cell lines and PGC-produced teratomas. The cells were identified by the enzyme phosphatase and the presence of a specific embryonic antigen (SSEA-1) that is also found in ES cells. Similar experiments utilizing the stem growth factor, along with LIF and the FGF, produced a PGC-derived pluripotent stem cell line that formed teratomas when injected back into mice[51].

Pluripotent stem cells were subsequently derived from PGCs of advanced human embryos[52]. Embryonic gonads containing PGCs were obtained from 5- to 9-week-old embryos, the result of therapeutic termination of pregnancy. These cells were cultured on a mouse feeder cell line inactivated by gamma irradiation. The complex culture medium contained a combination of growth factors and the differentiation inhibitor (LIF). In culture, the PGC cell lines maintained a normal chromosome complement. Spontaneously, the cell lines formed multicellular embryoid bodies that produced a specific enzyme (alkaline phosphatase) and five groups of specific surface molecules (glycoproteins and glycolipids) previously used to characterize pluripotent ES and embryonic germ (EG) cells. Through the use of individual antibodies produced to recognize cell components of specific cell types, early differentiating cells found in all three EG layers were detected in the embryoid bodies. As seen with the mouse ES and EG cultures, the outer layer of the embryonic body reacted with a protein (alpha-feto protein) identified with primitive gut and embryonic membranes. Other specialized cells recognized by specific antisera included premuscle (myocyte) cells, connective tissue supporting cells, primordial blood vessel cells, precursor neural cells, and surface epithelium. These immunological markers, associated with recognizable cells suggested that the human EG

cells are pluripotent. In both the ES and EG cell lines, the next major hurdle will be to actively direct stem cell culture into specific cell types.

### Testis Stem Cells and Transgenesis

Most transgenic experiments are the result of introducing DNA into fertilized eggs or by transfection of ES cells that are later incorporated into blastocyst stages. The efficiency of these germ-line transmissions of the introduced gene runs from 4–10%. As noted earlier, in the mammalian testis a small number of the spermatogonial cells are reserve stem cells (about 1%) that can self-renew and also serve as a source of future precursor spermatocytes that differentiate into mature sperm. A different approach to transgenesis has been taken by Brinster[53]. The fundamental procedure is to introduce a retrovirus containing an integrated mouse gene into gonial stem cells either directly while in the testis or after harvesting germ cells from a fertile male adult. The transduced stem cells have a marker reporter gene that stains the progeny of the transfected cells blue. The modified cells are then transplanted into the testes of nonfertile male hosts. Since only the introduced stem cells take part in the regeneration of mature sperm (spermatogenesis) in the host animals, those animals containing the transduced gene in their testes will exhibit blue patches in the tubules of the testes. When these animals were mated with a normal female, some of the offspring (both male and female) contained the transduced gene and in the males, all of the testes stained blue. The gene was incorporated into specific sites in chromosomes in about 4.5% of the progeny[54]. The same reporter gene can be introduced into 2–20% of cultured spermatogonial cells with similar results. Analysis of the integrated retroviral vectors shows that insertions into the host DNA (southern blot analysis) occurs at diverse sites on different chromosomes. Furthermore, expression of the inserted gene was maintained for three generations. In these cases, the same chromosome site of a father and his progeny or of a mother and her progeny was retained. Transduction of male gonial stem cells is another avenue for germ-line changes in many species.

## *Blood (Hematopoietic) Stem Cells*

Another group of cells found in the embryo are pluripotent blood stem cells that ultimately form diverse types of blood cells: red blood cells and various types of white blood cells (monocytes, granulocytes, cells that form the blood platelets) and cells of the immune system: “B” cells (antibody producers) and “T” cells (cellular immune response). Each of these cell types is derived along eight separate differentiation pathways in the process of blood cell formation (hematopoiesis).

In mammals, embryonic hematopoietic stem cells originate initially from mesodermal “blood islands” of an external embryonic membrane, the yolk sac. Later hematopoietic stem cells are found in mesoderm of the embryo located on a membrane adjacent to the major aorta artery. This shift in the site of blood stem cell populations was shown when cells from the latter region were taken from quail embryos and transplanted into the yolk sacs of chick embryos. The chick embryos later developed blood cells from the transplanted stem cells that were identified as quail blood[55]. Evidence from experiments on mouse embryos indicated a similar origin. Later, the stem cells for red blood cells and most of the white blood cells appear in the liver, then the source of the resident stem cells moves sequentially to the spleen and the bone marrow. The successive replacement of new cells with the same function are collectively called isoforms. In the chick embryo, some precursor stem cells will migrate from the yolk sac into the developing thymus gland and other lymphoid areas where they form lymphocytes of the immune system[56]. In the adult, hematopoietic stem cells will be found in the bone marrow as well.

As the origin of the stem cells for the various types of blood cells changes, there is also a progressive change in the cellular products or isoforms as the embryo prepares for postnatal development. An example can be seen in the red blood cells. During embryogenesis, development of erythrocytes (red blood cells) proceeds from precursor erythroid cells that ultimately eject their nuclei and become terminally differentiated red blood cells. The primary differentiation product of the red blood cells is hemoglobin, the oxygen-carrying molecule that demonstrates how activation of different genes can change the nature of hemoglobin as the embryo develops. These modifications also parallel the shift in the production sites for red blood cells from the external yolk sac to bone marrow of the fetus. In the human embryo, first an embryonic form of hemoglobin is produced in the red blood cells and then a fetal form of hemoglobin is found in the red blood cells as the site of synthesis moves into the spleen and liver. By the time of birth, adult hemoglobin replaces the fetal form at the same time that the production of the stem cell–derived red blood cells has shifted to the bone marrow[43]. As each cell type evolves through differentiation, its cell's function can change through adjustments by the controlling genes.

## ADULT STEM CELLS

### Regeneration from Reserve Adult Stem Cells

One form of regeneration involves the continuous production or self-renewal of cells as just seen in the process of hematopoiesis. A progenitor cell divides and reproduces itself, but also has the capability of forming into a precursor cell for one or more differentiated cells. This form of regeneration is *physiological* regeneration whereby certain cell types are replaced as a result of normal cell depletion. In older embryos and certain adult tissues, there are partially differentiated progenitor cells or "stem cells" that divide and give rise to a specific progeny or stem of new cells[57]. These cells provide an actively growing cell reserve that serves as a source of replacement cells. Adult stem cells that replenish cells lost by physiological turnover are evident in separate areas involving progenitor cells for the epidermis, the small intestine, the stomach, and the blood. The latter production sites were described for the embryo and a similar series of cells occur in the adult bone marrow.

#### *Adult Hematopoietic Stem Cells*

Throughout the adult life, the bone marrow serves as a repository of primary stem cells that produce separate hematopoietic stem cells for each of several blood cell types, not unlike the process of blood formation found in the embryo. It is also the source of mesenchymal stem cells for a variety of cells associated with the body support systems. A seminal experiment by Till and McCulloch[58] came when they transplanted normal mouse bone marrow into lethally irradiated mice in an attempt to repopulate the destroyed bone marrow hematopoietic stem cells. After transplantation of the normal marrow into the irradiated site, the mice recovered. Examination of the spleen revealed many sites called colony forming units (CFUs) that were derived from stem cells in the implanted bone marrow. Each colony consisted of diverse blood cells, red and white cells, and precursor cells for the blood platelets. Significantly, they were able to transplant stem cells from the colonies and produce secondary stem cell colonies.

In mammals, the evidence indicates that the developmental origin of cells in the blood is from a self-renewing stem cell that can be recovered from whole bone marrow or peripheral blood. Generally, this is done by the use of a clone of identical antibody molecules (monoclonal antibody) each for an identical antigen site found only on early progenitor cells, in combination with one of several cell-sorting techniques used to separate the stem cells. The result is a common "pluripotent" blood stem cell that activates a hierarchy of stem cells (Fig. 10). Under the influence of the stem cell factor, the "pluripotent cell" produces a pair of multipotent stem cells: (1) a lymphoid stem cell precursor and (2) a myeloid stem cell precursor. The lymphoid stem cell directly produces intermediate progenitor differentiating cells that either become the T cells (antibody producers) or the plasma cells (cell immune response) of the immune system. The myeloid stem cell produces three distinct multipotent stem/progenitor cells with a more limited potential and restricted self-renewal that are committed to six specific lineages[33]. They continue to differentiate in the presence of various growth factors and to date at least eighteen separate cytokines have been identified regulating this process. The first of these is a lineage-limited precursor that can only form one cell type, the erythroid (red blood cell) progenitor. It, in turn, passes through three intermediate precursor cell differentiation stages that end in a terminally differentiated red blood cell.

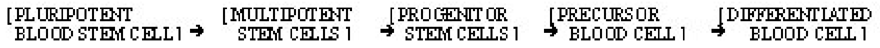

**FIGURE 10**

A second committed progenitor cell will differentiate into a mammoth MEGA-sized cell responsible for producing blood platelets, which are fragments of the MEGA cell that function in blood clotting. The third stem/progenitor is the granulocyte-macrophage cell, a multipotent stem cell that leads to a precursor for white blood cells or leucocytes (neutrophils, basophils, and eosinophils) that forage for cell debris or bacteria, secrete special products, and function in allergic immune responses. Still another monocyte precursor cell terminates in large scavenger cells called macrophages[33]. The final eight differentiated cell types are derived from stem cells along their own developmental pathway (Fig. 10). Once a particular pathway is chosen, the transition between stages is controlled by separate groups of growth stimulators and inhibitors.

Verification for the stem cell hierarchy came when hematopoietic stem cells were isolated from the fetal liver of mice[59]. By the use of cell surface markers, a primitive self-repopulating cell capable of reconstituting the entire hematopoietic heritage was recovered. From this cell, a pluripotent hematopoietic stem cells produced, in turn, the myeloid stem cell and an intermediate stem cell and other committed progenitors leading to differentiated cells of the myeloid stem[60]. The accessibility and their potential use as a source of related tissues has broadened the use of hematopoietic stem cells for related tissues. Recovery of the stems cells has been expedited with the discovery of a monoclonal antigen and antibody (CD34) by Civin and Small[61]. The antibody binds to the antigen on the stem cell surface separating them from other blood cells. In addition to their use in research, clinically the stem cells have been used to re-energize the immune system after cancer therapy.

## *Epidermal Stem Cells*

Other cells of the adult organism are in a constant state of renewal to replace cells that are lost, used up, or shed by the organism. This natural replacement or physiological turnover includes cells shed from the epidermis of the skin. These cells are fully differentiated (terminal differentiation) to perform specific functions. The nonspecialized areas of the skin, i.e., nonglandular, known as the epidermis, consists of outer (keratinized) layers of cells that are constantly moving to the surface where the dead outer layers are shed. Keratin granules are fibrous protein deposits characteristic of these layers. The keratinized layers cover a transitory layer of prekeratinized cells lying over an innermost basal layer. The basal layer is the source of dividing cells, some of which are immortal stem cells. Some of their progeny self-replicate as stem cells while others become committed progenitor cells and move into the prekaratinized layer, thus constantly replacing the outermost lost cells. The cells found in the deeper layers of the skin (basal layer) are constantly being tapped to replace epidermal cells lost from “wear and tear”. The stimulus for their differentiation is a specific growth factor. These precursor cells may also take up pigment from resident pigment cells found in the same basal layer that came from the embryonic neural crest.

Regeneration of the skin by skin grafts is dependant on renewal of stem cells in the graft. Recently, Pelligrini [61a]and colleagues isolated keratinocyte stem cells by mean a cell marker (p63) that has enabled attempts to regenerate skin by tissue engineering [61b]. The isolated keratinocyte stem cells are amplified in culture and grown into an epidermal sheet. A separate artificial dermal scaffold is then seeded with fibroblasts. The two layers are combined in their normal orientation and applied to skin wounds with some success. A limiting factor has been the ability to replenish the stem cells in the graft.

Similar stem cells are found in the lining (epithelium) of the digestive tract. The inside of the small intestine has very small finger-like projections (villi) projecting into the gut cavity. These projections are covered with a single layer of cells forming the epithelium. The latter extends down the villi into deep clefts (crypts) between each villus. The epithelial cells on the villi are highly differentiated mucus cells and cells for absorption of nutrients. Ultimately, the cells die, are shed, and need to be replaced. Deep in the clefts are partially differentiated stem cells, capable of forming new stem cells, but are also the source of progenitor cells of terminally differentiated cells on tips of the intestinal villi. The overall population of intestinal cells remains constant so that the cells replaced equal the number of cells lost and maintain a “steady state” population[62].

## Stem Cells and Regeneration in the Liver

During embryogenesis of the liver, multipotent stem cells from the endoderm migrate into the liver primordium. There they form hepatoblasts, embryonic precursor cells that have dual differentiation options. In turn, they differentiate into bipotential progenitor cells, either forming into hepatocytes, the major secretory cell of the liver, or epithelial cells of the bile duct. In normal cell maintenance of the adult liver and even after induced cell loss, replacement of hepatocytes and bile duct epithelial cells is accomplished by proliferation of resident differentiated cells. Stem cells do not seem to be involved during normal repair and self-renewal[63].

The liver of higher vertebrates can regenerate when part or a whole liver lobe is lost by surgery or from other trauma. Replacement of the liver mass is done by an explosive proliferation of the original liver cells (compensatory hyperplasia). The original structures do not grow back, but the individual cell types proliferate under stress resulting in overgrowth or hyperplasia from an increase in cell numbers. All of the cells making up the liver, hepatocytes (the central functioning liver cell), bile duct cells, endothelial cells of the blood sinuses, Kupffer cells, etc. are all affected, and are orchestrated by a variety of growth factors and hormones[64]. Each of the cell types is stimulated to replace itself. The hepatocytes provide the mitotic stimulus for the other cells. In fact, the hepatocytes dedifferentiate, proliferate as clones, and then differentiate into mature hepatocytes or a duct-like cell structure. Regeneration of the kidney, pancreas, adrenal glands, and ovaries follows a similar course of events.

However, the presence of liver stem cells, normally quiescent stem-like epithelial cells, emerged from studies on adult mice and rats. These stem cells are believed to form in the epithelium of smaller bile ductules. They appear when the regenerative ability of hepatocytes is inundated and can be found after chronic liver disease or when cancer has destroyed much of the liver[65]. When activated, they form bipotential progenitor cells, known as oval cells. Oval cells have the option of forming either hepatocytes or bile duct epithelial cells or they terminate from programmed cell death (apoptosis). There is also evidence that oval cells can form a “compartment” of other differentiated epithelial cells of the intestine and pancreas[63]. Numerous attempts have been made to isolate stem-like liver cells including oval cells. These poorly differentiated cells can be cultured and transplanted back into genetically identical and compatible (syngenic) inbred animals where they differentiate into hepatocytes. After severe liver injury, Grisham[63] postulates that facultative liver stem cells lead to the proliferation of oval cells. The normally inactive cells then proliferate and migrate through the liver, ultimately differentiating into hepatocytes and bile duct cells.

## Muscle Cell Regenerates

A different situation is found in external voluntary muscle (skeletal) cells of higher animals. During embryonic development, a group of cells originate from two bands of the mesoderm lying parallel to the developing neural tube. These cells are embryonic mesenchyme cells that give rise to progenitor cells of skeletal muscle, the myoblasts[46]. A number of myoblasts begin to fuse and produce a myotubule cell that is multinucleated and nondividing; the nuclei of the myotubule are located peripherally below the cell membrane (sarcolemma). Outside the cell is a thin, extracellular covering, the basal lamina, and just beneath it are some residual, nonfused myoblasts that remain as satellite cells. While muscle cells are normally nondividing, muscle regeneration was described in the amphibian newt[57] as a process that parallels a reverse of muscle fiber formation, i.e., individual nuclei and cytoplasm separate from the muscle fiber, then produce a mononucleate-dividing myoblast by the process of dedifferentiation. The reversal of the multinucleated myotubes from newts was also demonstrated when they were placed in culture and cells re-entered the cell-division cycle. Likewise, purified myotubules from the newt that were labeled with a lineage tracer and placed beneath the wound epidermis of an amputated limb, reversed their differentiation, proliferated, and were later detected in the wound bud (blastema), reappearing as labeled muscle cells[66]. There is a difference in muscle regeneration of newts vs. higher vertebrates. Experiments on mouse muscle cells in culture and transplantation of minced muscle in living animals[67] suggested a different sequence for the restoration of muscle. *In vivo* experimental models involving muscle transplantation, transgenic, and knock out mice have demonstrated that the completely differentiated, multinucleated muscle fibers can regenerate. However, the muscle fibers do not undergo dedifferentiation, but utilize special sequestered muscle precursor cells, the satellite stem cells.

## Muscle Satellite Cells

Satellite cells, the putative stem cells of muscle, are implicated not only in the growth, repair, and maintenance of skeletal tissue, but possibly in muscle regeneration of higher vertebrates. Normally they reside as inactive, nondividing cells, but can be reactivated when muscle is stressed or damaged. This initiates the release of mitogens and growth factors that activate the small, quiescent mononucleate satellite cells found adjacent to the muscle cell fibers. Early radioactive tagging experiments showed that they can be incorporated into muscle fibers of young, growing rat muscles. They differ in how they develop and decrease in number depending on whether they are obtained from prenatal muscle or later adult stages, and can retain the same characteristics of their origin if derived from “slow” contracting muscle or from “fast” muscle cells. Various markers for muscle cell components (myofibrils) and muscle cell adhesion molecules have been used to follow these muscle precursor cells into regenerating myotubules and mature fibers of muscle cells[68]. After their activation in the intact muscle, the satellite cells become muscle-generating (myogenic) precursor cells. A vast amount of evidence indicates that the satellite cell in adult muscle is definitely different from the ES cells[69,70]. The activation and subsequent differentiation of satellite cells are controlled by myogenic regulatory factors that promote their differentiation into myoblasts. The myoD family of four transcription factors, including myogenin, regulate both satellite cell activation and differentiation[70]. As stem cells, the satellite cells are also affected by growth factors (such as bFGF, PDGF [platelet-derived]) that stimulate proliferation and others that regulate proliferation: the colony stimulating factor (CSF-1), LIF, and TGF. Many of these factors are also chemoattractants to macrophages for both myoblast and satellite cells[69].

## Muscle Satellite (Stem) Cell Therapy

A collection of genetic muscle diseases, constituting muscular dystrophy, result in the degeneration and deterioration of function in skeletal muscle. This condition elicits muscle cell death, regeneration, and progressive degeneration of the muscle. The muscle cell is surrounded by the basement membrane that supports and links to the inner cell membrane. Several different defective molecules of the basal lamina have been implicated in muscular dystrophy: collagen type VI, dystrophin, and laminin. One component lacking in muscular dystrophy is an essential protein called dystrophin. Experiments on the MDX mouse, an animal model for human Duchenne muscular dystrophy, and a similar dog model showed that implantation of normal muscle precursor cells resulted in their incorporation into muscle fibers and the expression of the missing dystrophin protein. Several issues needed to be resolved, including overcoming rejection of the cells by the host immune response and the necessity of establishing a continuous presence of stem cells capable of producing the missing gene product[69].

In another study, muscle-derived stem cells were obtained from the MDX mouse with a "X" chromosome–linked progressive muscle degenerative disease[71]. The affected muscle cells again lacked the essential protein dystrophin. Clones of cells from slow-adhering primary muscle cells were inoculated with genes by the transfection procedure using a plasmid (a portion of DNA capable of independent replication) that contained genes for an enzyme (beta-galactosidase), minidystrophin, and antibiotic (neomycin) resistance. Following purification and clone isolation, a putative muscle-derived stem cell (mc13) line was established that was capable of differentiating into both muscle (myogenic) and bone (osteogenic) precursors either in culture or *in vivo*. These cells expressed three markers for myogenic precursors (desmin, c-met, and MNF), but also shared markers for hematopoietic stem cells and a marker shared by both skeletal and hematopoietic stem cells. After injection of the mc13 cells into muscles of the hind limb or into the blood stream, the marked cells contributed to muscle regeneration and partial restoration of dystrophin in the MDX mice. When the mc13 cells were directed into an osteogenic lineage (by the production of an osteogenic protein BMP-2), they were also found to enhance the healing of a skull defect in mice while nontransduced cells gave no evidence of healing. Thus, these muscle-derived stem cells exhibited a multipotential for both muscle cell formation and bone healing.

Another group of muscle cell–related basal membrane glycoproteins, called merosins or laminin, are found in the basement membrane that surrounds and supports muscle cells. They are involved in severe congenital muscular dystrophy, particularly in a defective form caused by the laminin alpha-2 gene. Defects of this molecule accelerate muscle degeneration and retard muscle cell regeneration. Several mutant ES cell lines with the defective laminin gene have been used to study muscle development, muscle cell differentiation, and regeneration *in vitro*[72]. It was found that the ES cells differentiate normally in culture, producing cardiomyocytes, myotubes, and smooth muscle cells. However, the mytotubes that form are unstable and die. It is believed that mutations in the gene for laminin alpha-2 may be partly responsible for muscle degeneration in merosin-deficient human muscular dystrophy *in vivo*. This group[72] is attempting to identify differentiation factors and mesenchymal stem cells that could be utilized as alternative sources in muscle regeneration.

A different approach to the cell therapy of muscular dystrophy was used[73] when arterial injection of mesodermal stem cells (mesoangioblasts) that were recovered from fetal blood vessels was made directly into muscle. These cells are capable of differentiating into most mesodermal cell types when manipulated by cytokines. The test organisms were dystrophic mice (alpha-sarcoglycan null), a mouse model organism for limb-girdle muscular dystrophy, which had the gene for alpha-sarcoglycan knocked out. Naturally recovered mesoangioblasts, radioactively labeled and traced with fluorescent DiI, were injected into the leg (femoral) artery of the alpha-sarcoglycan–deficient mice. These cells, unlike other muscle satellite cells, were capable of passing through the blood capillary wall (endothelium). After injection, the labeled mesoangioblasts were soon detected in the extracellular spaces of all downstream skeletal muscle, particularly in the areas of degeneration and regeneration. Fluorescent dye (DiI) experiments showed them capable of migrating long distances and being incorporated into regenerating muscle fibers, partially restoring the muscle form and function. The restoration of the alpha-sarcoglycan protein and other members of the glycoprotein complex was also found with the wild type angioblasts. Ultimately, similar fetal cells may be utilized for human applications after proper immune measures are developed.

## Heart Muscle Cells

In lower vertebrates, regeneration of the adult heart is possible as found in the amphibian newt[74] and more recently demonstrated in the zebrafish[75]. When 20% of the heart ventricle was removed, cardiomyocytes began to proliferate (along with myotubules), reaching a peak in 14 days when they migrated into the wound area. As in liver regeneration described earlier, there is no dedifferentiation. Complete cardiac regeneration is accomplished within 2 months.

The loss of heart myocytes in the adult mammal leads to reduced or lost function. For effective myocardial repair, engraftment, the inoculation of additional cells that infiltrate the tissue after cell insertion, has been used to bolster the number of myocytes in the adult mammalian heart. To enhance a source of pure heart cardiomyocytes, directed differentiation of totipotent ES cells was used to generate cultures of cardiocytes for intracardiac grafts[76]. Mouse ES cells were transfected with a fusion gene consisting of the muscle molecule myosin gene promoter (alpha-cardiac myosin heavy chain) and a Cana sequence that encoded for a related enzyme (aminoglycoside phosphotransferase). After cell differentiation *in vitro*, a pure culture of cardiomyocytes was produced. Purity of the myocytes was based on various cell immunological tests for myosin and electron microscopic analysis that showed typical cellular components of cardiomyocytes. The selected cardiomyocytes were then tested by injection into the heart muscle (myocardium) of the heart ventricle in dystrophic MDX mice. The fates of the engrafted donor cells were followed by applying antidystrophin and then detection by gene amplification of the muscle cell transgene for the myosin enzyme using the polymerase chain reaction, a procedure that allows molecular cloning of a specific DNA fragment by the use of an enzyme, DNA polymerase.

The engrafted regions revealed normal myocardial cells from the injected ES cells up to 7 weeks after implantation. While the cardiomyocytes from the ES cells provided an alternate source of donor cells for engraftment, the proliferation of the cardiomyocytes was still limited both *in vitro* and *in vivo*.

## *Mesenchymal Stem Cells*

In the embryo, there is a cellular hierarchy of closely related cell types that ultimately produce the body support (skeletal) system. The mesoderm-derived mesenchyme stem cells are progenitors for a family of precursors that become functionally committed as they progress into highly specialized differentiated cells. Separate embryonic precursor cells develop into bone-forming cells (osteoblasts), cartilage forming cells (chondroblasts), muscle forming cells (myoblasts), connective tissue precursors (fibroblasts), and fat producing cells (adipocytes). There are similar mesenchyme stem cells in the adult. A primary source of these multipotent stem cells was found in the bone marrow by Friedenstein in the 1970s[cited in 77]. Bone marrow stromal cells can be isolated and separated from the hematopoietic stem cells that also reside there. Such cells have been shown to be the progenitor cells for several mesenchymal tissues. Marrow stromal cells were taken from transgenic mice that had received a mutated gene for collagen I, one form of a fibrous protein that is assembled into collagen fibers found in a variety of tissues, connective tissue, bone, cartilage, tendons, ligaments, etc. After infusion of the genetically marked cells into mice that were X-irradiated, the cell progeny that contained the mutated collagen gene were first found in the bone marrow and the spleen of the recipient mice. Ultimately, the marked cells had contributed to mesenchymal derivatives such as cartilage and bone (but only collagen I was produced in bone) that demonstrated the multipotential of the stem cells[77].

### Bone Remodeling, Repair, and Regeneration

Intact bone in adult mammals is constantly being replaced by a continuous process of bone remodeling such that from 5–10% of bone is replaced each year. This process is similar to the repair process following bone fractures. Bone formation is provided by bone-forming cells (osteoblasts) that counteract bone destruction by other bone-destroying cells (osteoclasts). The primary function of the osteoblast is to synthesize and deposit an external matrix consisting of collagen fibers. Under the influence of a vitamin D–derived hormone, a gene is stimulated to produce another major protein, osteocalcin, in the matrix. This is followed by the precipitation of calcium phosphate during bone mineralization. The osteoblasts become surrounded by the bony matrix and persist as mature bone cells (osteocytes) that help maintain the bone. Bone formation is also under endocrine regulation. Indirectly, the hormone leptin, synthesized by fat cells (adipocytes), inhibits bone formation by fully differentiated osteoblasts through receptors in the major endocrine center located in the floor of the brain (hypothalamus)[78]. The role of leptin is far reaching since it not only controls bone mass, but also obesity and reproduction and underscores the complexity of stem cell differentiation.

Bone remodeling is regulated by specialized resorption bone cells originating from stem cells in the bone marrow. The stem cells differentiate into osteoclasts under the influence of many growth factors and hormones[79]. Similar to development of muscle cells, a macrophage becomes a multinucleated cell formed by the fusion of single cell hematopoietic progenitors into a large specialized osteoclast. Accessory cells produce a growth-stimulating factor (M-CSF) that causes the precursor cells to proliferate and a receptor for the activation of a nuclear factor (Rank) that stimulates osteoclast differentiation. A third protein, osteoprotegrin, regulates osteoclast production and thus the quantity of bone resorbed. In the process of bone resorption, the osteoclasts develop a ruffled border along which the cell releases acids to dissolve the mineralized bony matrix and hydrolases (cathepsin K) that digest the organic components. Bone resorption is also controlled through the production of the hormone parathormone from the parathyroid glands, plus vitamin D hormone and steroid sex hormones such as estrogen. It has long been known that a decrease in estrogen at menopause leads to osteoporosis.

Breaks or fractures in long bones of the limbs go through a discrete sequence of repair changes initiated by inflammation, then proliferation of cells that reside in the bone sheath (periosteum) and the formation of a fibrous-granular clot, the soft callus. The callus is gradually converted to a hard callus, first through the formation of cartilage by specific cartilage forming cells (chondrocytes) and then mineralization of cartilage by bone cells that leads to bone remodeling. This entire process is stimulated and controlled by a panel of growth factors (TGF, EGF, PDGF, and FGF). TGF and EGF initiate the reparative process while PDGF promote the scavenger cell (macrophage) activity. The TGF produced by chondrocytes and precursor bone cells stimulate local cell division, differentiation, and regulate collagen expression and cartilage synthesis. These are aided by a group of bone- and pattern-forming (morphogenetic) proteins (BMPs) that initiate differentiation of mesenchymal precursor cells for bone[80]. The growth hormone from the pituitary also plays a major role.

The differentiation of osteoblasts from stem cell–derived mesenchymal progenitor cells is also influenced by a group of growth factors: TGF-B, FGF, and Indian hedgehog (Ihh) factor. In addition, osteoblast differentiation is controlled by transcription factors, such as Cbfal, specific for differentiation of mesenchyme cells destined to become osteoblasts. Once differentiated, the osteoblasts are further controlled by the Cbfal transcription factor that regulates the gene Osteocalcin, which is only expressed in fully differentiated osteoblasts[78].

Experiments in animal model systems did demonstrate the capacity of skeletal stem cells to regenerate bone and related tissues (cartilage, adipocytes, etc.) in large segmental defects of long bones[61]. Skeletal stem cells (mesenchymal stem cells) were isolated from bone marrow and then their numbers expanded *in vitro* under conditions described previously. The stem cells were then allowed to attach to ceramic particles (hydroxyapatite/tricalcium phosphate). The composite was then transplanted into defective bone areas where the skeletal stem cell–vehicle regenerated a three-dimensional bone repair site. This included bone and stroma, adipocytes, and cells that support hematopoiesis.

The potential of human bone marrow–derived stem cells, mesenchymal stem cells, as been extended to therapy for osteogenic imperfecta (OI), a congenital disorder of mesenchyme cells in children This genetically inherited disease causes fractures, skeletal deformities, and inhibits bone growth. It is a result of a defective type I collagen being produced in bone. Clinical trials on children using bone marrow mesenchymal stem cells obtained from human bone marrow donors resulted in limited bone growth in children. These genetically unlike (allogenic) gene-marked mesenchymal stem cells were engrafted into patients where they differentiated into osteoblasts and skin fibroblasts without chemotherapy[81].

A somewhat different procedure for treating OI has utilized gene targeting of stem cells[82]. Mesenchymal stem cells were recovered from bone fragments of patients with OI following surgery. The bone marrow stem cells were first isolated and expanded in culture. They were then manipulated genetically by means of an adeno-associated virus *in vivo* to eliminate a dominant negative mutant. The gene targeted autologous (same individual) cells that were then tested for their ability to form bone in mice. First, gene-modified mesenchymal stem cell clones were induced to form osteogenic cells in culture and these cells were implanted into immunodeficient mice. Normal bone-forming osteocytes and bone matrix were found in the implants from the targeted clones. That the cells were human was verified with human-derived antibodies to human collage and mitochondria. The multipotential of the gene-modified mesenchymal stem cells was further shown by the induction of fat-forming adipocytes. The production of gene-modified mesenchymal stem cells showed that gene targeting is possible in adult tissue–derived stem cells and that similar clinical trials may be successful in humans.

### Adipose Cells

A possible alternate source of mesenchyme stem cells has been found in human adipose tissue[83]. Cells obtained from liposuction have produced a fibroblast-like heterogenous population of cells. The lipoaspirate-derived (PLA) cells were predominately cells of mesodermal or mesenchymal derivation, based on the presence of a filament protein vimentin, that were mixed with endothelial cells, pericytes (progenitors of blood capillaries and smooth muscle), smooth muscle cells, fibroblasts, prefat adipocytes, and a putative multipotent mesenchyme stem cell fraction. The specificity of the PLA component cells was determined through immunofluorescence of monoclonal antibodies unique to each cell type. Additional verification was obtained with similar cell surface markers with a cell-sorting technique (cell flow cytometry) to determine the quantitative measure of each class of cells. Lineage-specific differentiation factors were employed to direct the PLA cell cultures into fat (adipogenic), bone (osteogenic), cartilage (chondrogenic), and muscle (myogenic) cell lineages. Each of these differentiated cell lines was determined by microscopy and various histochemical stains, in addition to specific antibodies, e.g., adipogenic: oil Red O for intracellular lipids; osteogenic: the presence of alkaline phosphatase and a calcified matrix; chondrogenic: Alcian blue and formation of a cartilage-specific collagen II; and myogenic: a muscle specific transcription factor (MYOD1) and myosin heavy chain. The multipotency of the PLA cells to differentiate into specific cell lineages was aptly demonstrated, but could also be due to already committed progenitor cells or contamination by multipotent cells from elsewhere. While the specific cell types may be very useful as a cell source, the presence of a multipotent stem cell from adipose tissue still awaits confirmation.

## Evidence for Transdifferentiation of Adult Stem Cells

Transdifferentiation is the conversion of a differentiated specialized cell with specific traits (phenotype) into another differentiated cell type expressing a new set of characteristics[84]. The result is stable and irreversible and may include terminally or partially differentiated cells. It has thus been implicated in the putative expansion of the multipotency of certain stem cells.

### *Bone Marrow Stem Cells into Liver*

The mutipotential of bone marrow stem cells as precursors to cells of the liver has been supported by several *in vivo* studies. As noted earlier, liver oval cells (hepatic oval) normally proliferate following liver damage and are believed to develop into duct cells or liver secretory cells (hepatocytes). These stem-like cells will grow when hepatocytes fail to proliferate. A possible external source of the oval cells from bone marrow cells was hypothesized[85]. The approaches used were all preceded by a special protocol that inhibited hepatocytes and induced liver damage, a key to oval cell proliferation and proliferation in the host animals. The first experiment involved bone marrow transplants from adult male rats into lethally irradiated syngenic female rats that was followed by oval cell stimulation. Nine days afterwards, they found that some of the oval cells in the recipient females came from the donor male bone marrow as they exhibited the male Y chromosome. This was based on detection of a DNA product from the Y chromosome by the polymerase chain reaction. By the

13th day after transplantation, the maturing hepatocytes in the liver expressed the same Y chromosome signal, indicating they were derived from the bone marrow stem cells.

These results were confirmed by detection of a particular "say" gene on the Y chromosome using "*in situ* hybridization". This method originally made use of external radioactive nucleic acid marker probes, but more recently, probes are created by an identifying attached molecule (often a fluorescent dye) to locate specific DNA gene sequences on a single DNA strand located in the intact chromosome. In practice, a section of tagged RNA that is capable of binding to a specific portion of DNA, such as the "say" gene, is applied and its presence is detected by the identifying marker. The result showed positive liver oval cells with the Y chromosome gene in the female rats that had received bone marrow transplants accompanying stimulated oval cell production. The same effect was detected in the mature hepatocytes on the 13th day, again verifying their origin from bone marrow stem cells.

In a second approach, bone marrow cells from male rats having a specific enzyme (dipeptidylpeptidase) were transplanted into female rats lacking the enzyme. The same hepatocyte inhibition/liver damage was inflicted. If cells originally from the bone marrow donor cells could be found in the liver, they would be recognized by a red/orange staining caused by the active enzyme. Indeed, the enzyme was expressed on oval cells and on transitional hepatocytes in the liver of host animals that confirmed their derivation from the bone marrow stem cells.

Next, liver transplants were made between a group of rats, where the recipient strain possessed a specific fingerprint for a family of tissue-compatible transplantation enzymes (histocompatibility isozymes) that the donor Brown Norway rats did not express. By the use of an antibody to this group of enzymes, they found that some oval cells were derived from an outside hepatic source and others, negative to the enzyme group, were derived from the donor liver. The foregoing results suggested that under specific conditions, the bone marrow cells could be progenitors to different types of liver cells and potentially could be used in therapy of acute liver trauma.

Similar experiments indicated that adult human liver cells can be derived from bone marrow. Donor bone marrow cells were found in the liver of female patients who had received a bone marrow transplant from a male donor. The donor cells were identified by means of a DNA-specific probe for the Y chromosome of males. After the transplantations, Y-chromosome-positive epithelial cells were found in the liver that were identified as hepatocytes by their expression of a signature molecule, cytokeratin 8. These results suggested that adult human hematopoietic stem cells could also provide a source of hepatocytes after liver damage[86]. Differentiation of bone marrow cells into differentiated cells of the liver has also been reported in humans[87].

The previous examples on bone marrow–derived stem cell nuclear plasticity in the liver was further examined in bone marrow–derived hepatocytes (BMHs) of mice[88]. The host mutant (Fah) mice were irradiated and bone marrow cells from genetically distinct (Fancc), homozyous mutant (duplicate forms for the same gene) donor mice were transplanted into the hosts. After the appearance of BMHs in the repopulated livers, the BMHs were serially transplanted in two successive procedures to new Fah mutant host mice. Instead of retaining their original paired dominate identical allele (homozygote) donor genotype, the derived liver hepatocyte chromosomes showed alternate paired genic forms (alleles) indicative of a disparate mixed genetic origin (heterozygote) when tested with a labeled probe for a genetic marker. While the liver was repopulated with up to 50% of new hepatocytes, the analysis did not support transdifferentiation of the donor stem cells into hepatocytes. Secondly, a chromosomal analysis was made of hepatocytes transplanted from female donor mice into male hosts. The cells contained from two to four multiples of the normal chromosome karyotype including the sex chromosomes XX and XY, a clear indication of hybrid cell fusion. It was concluded that most of the hepatocytes from the bone marrow had formed by cell fusion between donor blood cells and host hepatocytes and not from differentiation of bone marrow stem cells.

## Bone Marrow Stem Cells into Neurons

Adult cells from mouse bone marrow stem cells also appeared to have the potential to form neuron-like cells. The first of two separate reports[89] indicated that normal adult mouse bone marrow stem cells transplanted into neonatal mouse recipients can migrate into the brain and then differentiate into cells with neuron characteristics. The host female recipients were specific "knock out" mice (Pu.1) that lacked many of the cells of the immune system (macrophages, neutrophils, B & T cells, and others) and were destined to die without a bone marrow transplant. The bone marrow donor cells, that lacked any evidence for neurons or glial cells at the time of transplantation, contained markers for neurons and further identified by the Y chromosome of males. This was done with a nuclear marker, NeuN, that fluoresced red after immunostaining while the Y chromosome was visualized by a green fluorescent conjugate. A second neuronal marker (NSE) was restricted to the cytoplasm. Transplant recipient mice were examined from 1–4 months after injection of the bone marrow cells. Bone marrow–derived Y chromosome cells were found in some cells from all of the host-transplanted mice. Furthermore, the bone marrow cells after migration into the brain had differentiated into cells expressing neuron-specific antigens in up to 40% of the cells examined after 4 months. Brains from unaltered females and males were used as controls and showed no Y chromosome staining or specific nuclear staining. These results raised the possibility that ultimately, bone marrow cells could be an alternate source of neural cells in the treatment of neurodegenerative diseases.

A simultaneous report of the versatility of adult mouse bone marrow cells was based on the investigations of Brazelton et al.[90]. Bone marrow–derived cells were genetically manipulated in transgenic mice to express a green fluorescent protein (GFP). These bone marrow cells were injected into other adult mice hosts that had been lethally irradiated to destroy their indigenous bone marrow. Initially, dissociated cells recovered from the brain of the host mice 1–6 months after inoculation and counted by cell sorting, showed that up to 20% of the fluorescent marked cells no longer expressed cell surface markers of bone marrow cells, but now exhibited neuron-specific proteins. Through the use of a noninvasive optical scanning microscopy that detects images at multiple levels to produce a three-dimensional image (laser scanning confocal microscopy), they were able to analyze GFP-positive cells in tissue sections that had originated from the bone marrow and migrated into the brain. Using fluorescence-labeled antibodies to neuronal proteins (NeuN) and a neurofilament (NF-H) protein, both proteins could be visualized by their unique fluorescence in the green fluorescent bone marrow–derived cells. Only a small number (0.3%) of up to 80,000 cells analyzed expressed neuronal markers. Moreover, markers were identified simultaneously in the same cell along with a third marker, Tuj1. This and evidence for the activation of a major signal transduction pathway characteristic of neurons indicated these cells were expressing genes specific to neurons, that confirmed the capability of bone marrow cells as a possible source of neuronal cells.

However, other types of experiments have failed to substantiate the universality of bone marrow transdifferentiation into neural cells. When mouse cells carrying the LacZ gene as a marker in donor cells were transplanted into irradiated C57 mice that were bone marrow deficient, the recipient hosts reconstituted blood cells that were 80–95% positive for the LacZ gene. There was no evidence for transdifferentiation into host neural cells up to 4 months after transplantation[91]. In another extensive study[92], chimeric animals were produced by transplantation of the expanded cells from a single GFP-marked hematopoietic stem cell into irradiated mouse hosts. While the progeny from the marked stem cells appeared plentiful in the white blood cells of the irradiated host, they found little evidence for transdifferentiation in the brain or in kidney, gut, liver, and muscle.

## Muscle Satellite Cells from Bone Marrow

Strong evidence exists that skeletal muscle satellite stem cells are not only the precursors of mature muscle fiber, but could be replaced by bone marrow–derived stem cells[93]. A two-stage conversion from bone marrow–derived stem cells to muscle satellite cells was made and, in turn, their transfer into myoblasts also necessitated sequels of trauma or stress. Mice were exposed to total body irradiation to destroy the bone marrow. Then, new bone marrow cells labeled with GFP were introduced. Using a series of fluorescent-labeled markers for nuclei, DNA, and specific muscle adhesion protein markers (integrins), it was found that the labeled bone marrow–derived cells replaced up to 5% of the satellite cells in the muscle cells. These new satellite cells remained inactive up to 6 months, but after the animals were given exercise induced stress, the GFP-labeled cells contributed to the regeneration of mature muscle fibers. In tissue culture, the satellite cells formed premuscle (myogenic) clones and when treated with muscle cell inductive media, they formed multinucleated muscle cells. When they were injected into muscle tissue, these myogenic cells also contributed to muscle fibers.

### Hematopoietic Stem Cells from Muscle

Several studies have suggested that putative stem cells can be isolated from muscles of adult mice and that they also can be multipotent progenitors for hematopoietic cells[94]. Skeletal muscle cells harvested from adult mice were briefly cultured and then mixed with identifiable whole bone marrow cells. The cell mixture was then inoculated into lethally irradiated mice. The bone marrow cells were used to provide sufficiently committed hematopoietic cells to rescue the bone marrow from the effects of lethal irradiation. After 6–12 weeks, the peripheral blood was tested and all animal recipients displayed each of the major blood groups with distinct muscle cell lineages. The incidence of muscle-derived blood cells was 10–14 times higher than those from whole bone marrow. They also found that a subpopulation of cells cultured from skeletal muscle cells shared characteristics with bone marrow–derived hematopoietic stem cells. These cells appeared to lack myogenic regulators and may have responded to environmental cues.

### Hematopoietic Stem Cell Chimeras

Adult bone marrow–derived stem cells can be integrated into mouse blastocysts. Mouse bone marrow hematopoietic stem cells from an adult were injected into preimplantation blastocysts, where they survived and generated a chimera of embryonic/adult hematopoietic cells. As the embryo developed, the donor cell progeny were found in the yolk sac, the fetal liver, peripheral blood, and later in the bone marrow of the adult. At the same time, they expressed an embryonic/fetal type of the blood hemoglobin gene[95]. In a converse experiment, suspensions of cells from transgenic mouse embryos with human beta-globin genes were injected into lethally irradiated mouse adults. Subsequently, spleen cell colonies (CFUs) from adult mice were examined for human beta-globin genes by DNA, RNA, and transcription analysis. The embryonic and fetal hematopoietic progenitor cells transplanted into the adult mice did transcribe the adult human globin gene. They concluded that the developmental stage of the hematopoietic cells controls the developmental fate of the transplanted progenitor cells.

After injury to heart muscle (myocardial infarction), resident heart stem cells will migrate to the site of damage where they differentiate into various heart associated cell types. However, these cells are usually insufficient to repair the damage. An alternate source of adult stem cells for treatment of myocardial infarction has been found in a subpopulation of hematopoietic stem cells[96]. First was a separation of a special subgroup of blood stem cells from mice that were then injected into the coronary artery of bone marrow–impaired irradiated mice. The cells were able to migrate and engraft into the damaged cardiac muscle and blood vessels where they differentiated into heart muscle cells (cardiomyocytes) and endothelial cells of the blood vessels. Another study employed the transplantation of highly purified stem cells from bone marrow into heart lesions of mice[36]. Bone marrow cells from male transgenic mice were harvested and stem cells were first separated from the heterogenous cell population according to cell size and density by a special centrifugation technique (counterflow centrifugal elutriation). Then the differentiated blood cells (lymphocytes, granulocytes, myelomonocytes) were removed with antibodies directed against their lineage-specific cell markers. The remaining cells constituted a targeted cell fraction having a deficient lineage (Lin-). Some of these cells marked with a GFP and having a specific stem cell surface marker (c-kit+) were sorted by specific antibodies. Hours after an induced heart infarction of young female mice, the marked (Lin-, c-kit+) bone marrow stem cells were injected into healthy tissue adjacent to the lesion in the heart ventricle. Within 9

days, newly formed myocardial tissue replaced 68% of the cells in the infarction site in 40% of the injected mice. The majority of the cells were new myocytes, but they also included endothelial cells, smooth muscle cells, arteries, arterioles, and capillaries. Mice treated with the stem cells also demonstrated functional heart improvement in their ventricular heart pressure. Both of these studies provided evidence that bone marrow stem cells are capable of regenerating myocardial tissue by their migration, proliferation, and differentiation *in vivo*.

An unusual ability of either hematopoietic stem cells or muscle-derived stem cells to partially correct the muscular deficiency of the MDX mouse was revealed in experiments on irradiated mice. Stem cells were introduced by intravenous injection of either hematopoietic stem cells or a purified set of muscle stem cells. The technique involved sorting a subpopulation of stem cells identified by exclusion of a dye (Hoechst) and recovering the population of muscle stem cells by means of fluorescence-activated cell sorting. First, it was found that stem cell isolates could differentiate into myoblasts in culture and expressed a muscle molecule called desmin. After intravenous injection of the cultured stem cells into the irradiated mice, the desmin-marked cells were found in 9% of regenerating muscle fibers. The muscle stem cells also produced satellite cells in recently regenerated host muscle fibers, indicating that both hematopoietic and muscle-derived stem cells were equally competent.

In a separate experiment [97], transplantation of normal bone marrow or bone marrow–derived stem cells from young male animals reconstituted the female host hematopoietic cell population compartment in 12 weeks. Furthermore, it was found that purified hematopoietic stem cells containing Y chromosome nuclei delivered to the hosts by the blood system could be detected in 4% of the myofibers. Significantly, these cells also expressed a partial restoration of dystrophin normally lacking in the mutant mice. The possible clinical effect is that stem cells from either bone marrow or muscle could be disseminated through the circulatory system for muscle repair. The results provided more evidence that bone marrow–derived stem cells can differentiate into muscle cells. Thus, there may be a relationship between bone marrow–derived stem cells and other tissue-specific stem cells.

It is still not resolved whether muscle satellite cells are the same as muscle stem cells, whether muscle stem cells are the progenitors of satellite cells, or if they represent two different cell populations. It has been proposed that satellite cells represent a pluripotent stem cell population since they can generate both myoblasts and cells of the hematopoietic system. Since many stem cells have been shown to have an increased potential, a hypothesis has been projected that all differentiated tissues contain pluripotent stem cells. The specific cell type generated would be a result of their response to their microenvironment and to specific growth factors[70].

One of the rigors of scientific research is that results reported in the literature must be reproducible in other laboratories. Several stem cell researchers have indicated that some results, particularly those involving transdifferentiation of multipotent adult stem cells, such as bone marrow cells forming muscle, liver, or neural cells, have not been replicated[98]. This involved evidence for adult stem cells "escaping" their multipotent cell compartments and undergoing transdifferentiation when they are first incubated with ES cells. The altered stem cell origin is often established by the presence of original stem cell markers such as the Y chromosome. Other experiments have projected that cell fusion may be involved. Bone marrow cells from female transgenic mice, exhibiting GFP, were cultivated with mouse ES cells in a medium containing the cytokine IL-3[99]. Subsequently, the original ES cells were ejected from the culture after treatment with an antibiotic puromycin. The remaining green fluorescent cells were embryonic "pluripotent" stem-like cells. Under appropriate conditions, these cells were capable of expressing a variety of genes relating to cell types of all germ layers including neuronal cells. However, genetic analysis of the cells indicated they had double the number of chromosomes, and were probably a hybrid of the original ES cells and the bone marrow cells. These and other studies have suggested that spontaneous cell fusion may account for changing the potency of some stem cells and may not result from transdifferentiation of the original stem cells. The efficacy of the results in the prior stem cell experiments may still be significant, but may not be the product of expanded multipotency of adult stem cells.

## Neural Stem Cells

Earlier it was seen that fetal precursor cells isolated from mouse neural crest cells produce clones of stem cells that develop into impulse-conducting neurons and accessory neural glia cells and smooth muscle cells *in vitro*. The search for possible neural stem cells (NSCs) in the adult led to the recovery of cells from a region (striatum) of the adult mouse brain[100]. These cells were cultivated with several growth factors to stimulate cell division: EGF, bFGF, and NGF. The cells first produced a cell cytoplasmic filament (nestin) found in embryonic neuroepithelial cells. This cell skeletal filament protein is recognizable when coupled with a bright red fluorescent dye. The protein is found in NSCs and in precursor cells and later disappears when the precursors differentiate into neurons or supporting cells[101]. The brain also possesses multipotent precursors for other brain-supporting glial cells (astrocytes and oligodendrocytes)[100]. An insight into how neural crest stem cells can produce different cell types along their migration path *in vivo* came from studies on neural crest cells recovered directly from freshly isolated mammalian fetal nerves. That these cells were multipotent stem cells *in vivo* was ascertained when they were directly transplanted into the neural crest pathway of chick embryos. Afterwards, the implanted stem cells differentiated directly into neurons and glial cells[102].

The question of what dictates the cell destiny of the multipotent stem cells *in vivo* remained. One possibility was that once neuroblasts differentiated, they could send a signal to the remaining stem cells that would both inhibit neurons and promote glial cells[103]. At issue was how these cells can first produce neurons and then glial cells when they are exposed to various growth factors[104]. Initially, the neural crest cells formed neurons when exposed to growth factors such as BMP. Later, they formed nerve sheath (Schwann) glial cells. Based on this observation, neural crest cells were separated from isolated nerves and cultivated into stem cell clones. Both neural crest cells and neuroblasts produce gene-controlled linking proteins that can engage specific binding sites on other molecules (proteins) and change their function. One family of these gene-controlled coupling proteins (ligands), Notch and Delta, has this ability. When a soluble Notch receptor ligand Delta-1 was added to the culture of neural crest cells, it inhibited neuronal differentiation of the cells, completely blocking the effect of the growth factor BMP. Furthermore, after withdrawal of Delta, the remaining undifferentiated cells now developed into glial cells. This suggested that such ligands on neuroblasts may provide a heritable cell signal that favors the formation of glial cells.

### *Expanded Potential of Neural Stem Cells*

Except for the examples cited, identification and isolation of NSCs from adult tissues has been a recent finding[7]. Furthermore, adult NSCs appear to have a broader spectrum of activity than once thought. Typically these cells would form both supporting (glial) and impulse transmitting neurons[101]. NSCs were isolated from the forebrains of both adult and embryonic mice (ROSA26) that had a distinctive transgenic marker (lacZ) that encodes for a bacterial enzyme[105]. Individual embryonic or adult NSCs were grown in culture with the growth factors EGF and FGF. Some were allowed to differentiate into typical nerve cells. Other cultured NSCs were injected into mice of a different mouse strain (Balb/c) previously irradiated sublethally to kill most of their bone marrow cells. Five months after injection of the stem cells, it was found that the recipient (host) animals possessed both immature blood precursor cells in the bone marrow and mature blood (myeloid and lymphoid) cells derived from either embryonic or adult NSCs of the Rosa mice. These mature blood cells were positively identified since they exhibited donor neural cell marker proteins as well as blood cell marker enzymatic proteins found in blood cell precursors, indicating that the NSCs may have a broader developmental potential than they exhibit *in vivo*.

### *Integration and Differentiation of Neural Stem Cells*

The NSCs of the adult mouse brain appear to have an even broader, multipotent ability. When ES cells are cultivated as embryoid bodies, they can differentiate into a variety of cell types. Whereas NSCs normally generate neurons, astrocytes, and oligodendrocytes, the cultured neural cells formed muscle-like cells (myocytes) that contained muscle filaments (desmin) and reacted with molecular muscle components. Consequently, the ability of ES cells to provide inductive signals that would modify the differentiation of adult neural stems cells was evaluated. Adult NSCs from (ROSA26) mice that produce a specific bacterial enzyme (beta-galactosidase) were cocultivated with ES-derived embryoid bodies[106]. The enzyme served as a marker since it could be used to attack a sugar releasing a blue dye. Similarly, antibodies against the enzyme aided in its identification. The ES cells, sensitive to the antibiotic neomycin, were eliminated from the cultures and clumps of exclusively NSCs (neurospheres) developed. Next, they injected the adult mouse NSCs into the cavity of the protective membrane (amnion) of developing chick embryos for possible integration into the embryonic germ layers where they would fall under the inductive influence of the chick embryonic systems. These mouse NSCs were able to form chimeric tissues in the brain and spinal cord and further developed into embryonic mesoderm derivatives (somites, notochord) found in the kidney, lung, stomach, liver, and intestine of the developing chick embryo. Progeny from the injected cells were identified by release of the blue dye and by antiserum to mouse-specific cell surface molecules. Moreover, the new cell types expressed both mouse- and tissue-specific markers. Single adult mouse neural (ependymal) stem cells were also isolated directly from the brain, prelabeled with a fluorescent dye (DiI), and cultivated into neurospheres. They also became integrated and formed chimeric tissues when injected into the amniotic sac of chick embryos. Ultimately, dissociated cells from an adult neural cell–derived neurosphere were either aggregated with normal mouse embryo morulae embryos or injected directly into the blastocyst cavity of mouse embryos. After transfer into a foster mother, the embryos developed until the 11$^{th}$ day. The adult NSC mouse/chick chimeric embryos, identified by reverse transcription polymerase (RT-PR) and the presence of the enzyme galactosidase, were located throughout the embryo. Again, blue-stained cells were found in the embryonic brain and spinal cord, and all derivatives of the embryonic germ layers, including liver and heart. When intact neurospheres were injected directly rather than merged with morula embryos, 12% of the survivors were positive. Notably, blood derivatives from the neurospheres were absent from both the chick and mouse embryo chimeras. NSCs thus appear to have a broad multipotent capacity. More importantly, this indicates that adult (neural) stem cells can be redirected back to their origin as ES cells and are potentially pluripotent when influenced by the inductive effect of the developing embryo.

## Stem Cells and Nervous Diseases

There are a variety of better-known diseases affecting both types of nerve cells in the brain and spinal cord.. Since most areas of the brain have few available stem cells, ES cells in particular[107], derived embryoid body cells, fetal stem cells, and other stem cell lines are the primary source of stem cells for study. Neurons that receive and process information often affect actions via a chemical transmitter. Several diseases are currently being investigated, such as Parkinson’s disease caused by a defect in the production of dopamine, a brain chemical. A related disease (Huntington’s) causes a similar, more specific, and localized defect of dopamine neurons. Other degenerative neurons in Huntington’s disease affect skeletal muscle cell control of movement. Another, Lou Gehrig’s disease or amyotrophic lateral sclerosis (ALS), causes damage to the motor neurons of the brain and the spinal cord. Still another disease, multiple sclerosis (MS), is both an autoimmune disease and a defect of nerve cells.

The glial cells, those that provide structural support, functional and guidance information to the neurons, are affected in MS that causes degeneration of the protective covering around neurons. Direct damage to the spinal cord has also prompted studies on stem cells for nerve cell regeneration. The usefulness of various scientific investigations directed toward stem cell therapy in the nervous system is discussed next.

## Directed Potential of Neural Stem Cells

Previous experiments with rodent-derived ES cells from the brain indicated that they could form multiple types of stem cells. However, selection of specific cells by controlling the direction of differentiation was not automatic. Certain glial cells (Schwann cells), a specialized nerve cell, produce a fatty protective sheath called myelin that insulates the cell (axon) extensions of nerve cells that are found in the peripheral nervous system throughout the body. Similar glial cells, oligodendrocytes, produce myelin sheaths around axons within the brain and spinal cord. Since this axon coating is necessary for normal function of nerve cells, a method for directing differentiation of glial cells was sought. ES cells from mice were cultivated *in vitro* into embryoid bodies under conditions that promoted neural cell precursors. When various growth factors (FGF, EGF, PDGF) were present, they favored the proliferation of glial precursor cells. When the growth factors were withdrawn, the cells developed into glial cells (oligodendrocytes, astrocytes) that produce a protein (CNP) found in myelin. The latter cells were then transplanted into the spinal cords of fetal and neonatal rats that harbored a myelin-deficient genetic disorder wherein they lacked the ability to form a myelin fat-protein complex (PLP) similar to a human genetic disorder. Two weeks later, myelin sheaths were found on the neurons. The glial cell precursors were also inoculated into the brain chambers of myelin-deficient 17-day-old rat embryos. At 3 weeks of age, PLP-positive nerve sheaths were found on neurons in various regions of the brain, indicating that glial cell precursors can be directed to differentiate into cells that can interact *in vivo* with host neurons by supplying the protective myelin sheath. This procedure may ultimately be the basis for the treatment of human neurological diseases[108].

Another approach to spinal cord injuries involving the myelin sheath came from the use of mouse ES cells that were induced to form immature nerve cells. Embryoid bodies were developed in culture and the neural cell aggregates were then injected into the hind limbs of adult rats that had sustained lower spinal cord injuries, 9 days after the induced contusions. After 2–5 weeks, the implanted cells identified by fluorescent antibodies had spread through the injured spinal cord and had formed into neurons and supporting astrocytes and dendrocytes. After 1 month, the rats demonstrated improved hind-limb weight support and locomotor coordination not observed in the control animals[109].

A similar report tested the ability of a human pluripotent stem cell line in a rat model for ALS. A human gonadal primordial cell line from an aborted fetus was the cell source. Gearhart and coworkers[110] introduced the cells into the cerebral spinal fluid within the spinal cord of rats that had been injected with a virus derived from primates, the Sinbai virus. The host animals had their motor neurons destroyed by the virus that simulated the damage caused by the disease ALS. Three months after inoculation of the PGC cells, many of the rats exhibited clumsy locomotion in a partial recovery suggesting that new motor neurons had formed. Others postulated that neuroprotective factors, rather than new neurons, might have been the recovery factor.

Evidence for similar cells was found in the adult human brain. Neural precursor cells from the adult brain, in a layer (subventricular zone) adjacent to the brain cavity, were recovered at the time of surgery. Cells identified by the neural precursor cell nestin were placed into culture and cell clones were developed in the presence of EGF and bFGF. In culture, the cells exhibited antigenic profiles of neurons and glia-like cells. When these cells were transplanted into adult rat spinal cord whose neurons were devoid of the myelin sheaths, the nerve axons were remyelinated and nerve impulse conduction restored[111]. These findings suggested that a common neural progenitor cell may be present in the human brain similar to those described for embryonic NSCs in the mouse and the rat.

Directed differentiation of the multipotent neural crest stem cells is also possible *in vitro*. It was found that cells cultured with a glial growth factor will promote differentiation of glial cells while the application of the BMPs favor neuronal differentiation[103]. Another growth factor, the ciliary neurotrophic factor, acting on the multipotent stem cells preferentially directs them into astrocytes. Similar instructive differentiation of neural crest stem cells occurs in the presence of the growth factors BMPs and EGF[101].

## Reprogramming Neural Stem Cells

Multipotent stem cells with extended potential and self-renewal are evident in the embryo, adult, and possibly other tissues. The sequence of cells forming from a NSC leads either to a neural progenitor cell or a glial progenitor cell that are usually limited in their potential to form alternate cell types of nerve cells. The neural progenitor differentiates into an impulse-transmitting nerve cell (neuron) while the glial progenitor may form a committed precursor oligodendrocyte cell (OPC) or an astrocyte precursor cell, both destined to form oligodendrocytes or astrocytes, respectively (Fig. 11). The glia are an ancillary group of cells that protect, monitor, and serve the neurons. However, glial precursor cells can dedifferentiate into neural precursor cells. When OPCs were[112] isolated and purified from the eye (optic) nerve of newborn rats and placed in culture with PDGF, it promoted cell proliferation and inhibited their differentiation. When PDGF was withdrawn and replaced with bFGF, more than 90% of the cells differentiated into oligodendrocytes. Alternatively, the OPCs could be directed into astrocytes by culturing them only in fetal calf serum, a growth enhancement fluid used for conventional tissue culture, plus PDGF for 3 days. Subsequently, if both growth stimulators were replaced with bFGF, more than 40% of the cells continued proliferation for several weeks and eventually resembled OPCs.

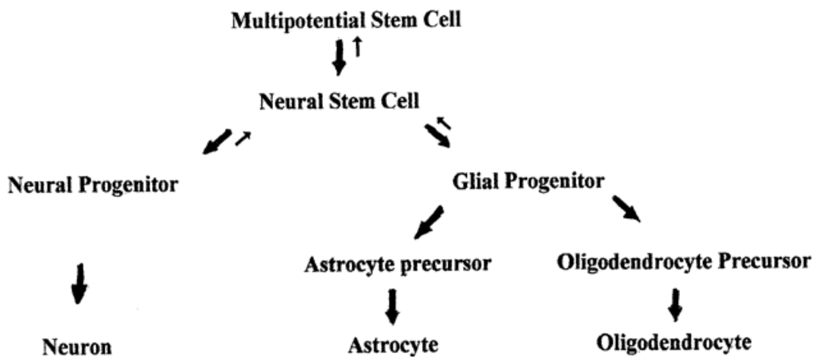

FIGURE 11

Certain characteristics of these cells suggested their potential had been broadened into multipotent NSCs. To test this hypothesis, purified OPCs were induced into astrocytes with fetal calf serum and PDGF and then cultivated them in bFGF alone from 2 days up to 1 month. After 2 days, over 40% of the cells reacted with fluorescent-tagged antibodies for a neuron-specific cytoskeletal component (MAPS), whereas less than 40% of the cells reacted with markers specific for astrocytes. Cells cultured for 2 months in bFGF now expressed additional neuron-specific markers suggesting that these cells had begun differentiation into neurons. When these cells were switched to PDGF alone, the new markers were enhanced. Further tests showed the cells possessed a mRNA for an enzyme needed for the synthesis of a nerve neurotransmitter GABA. This supported the prior evidence that neuronal cells could differentiate from the precursor OPC cells. Next the researchers asked whether the cells grown in bFGF for 2 months could still form oligodendrocytes. To do so, they removed the bFGF and added PDGF and a thyroid hormone known to encourage their growth. After 5 days, 30% of the cells were identified as oligodendrocytes by a specific antibody. In the same manner, they tested to see if any of the cells grown in bFGF for 2 months could form astrocytes. By removing bFGF and adding PdGF plus BMPs, about 10% of the cells became astrocytes within 5 days. The OPCs do appear to have been reprogrammed and returned to a NSC state. However, the option still remains that the cultures still retained multipotent stem cells.

In the adult rat brain, the hippocampus has a normal population of NSCs that are believed to give rise to either astrocytes or to neuronal progenitor cells[113]. These adult NSCs from the rat brain, precursors of neurons and supportive glial cells (oligodendrocytes and astrocytes), are also dependant on growth factors and other external stimuli to promote their specific fate and proliferation. NSCs were harvested from a specific brain region, the hippocampus of postnatal rats, to promote neurogenesis of adult NSCs[114]. The cells were cultured and then modified with a retrovirus so that their genome expressed GFP+. These cells, dependant on the growth factor FGF-2 can be induced to form and promote proliferation of all three neural precursors, each recognized by cell markers specific for neurons, oligodendrocytes, or astrocytes. Such cells do require additional stimuli. Accordingly, when cultured with a feeder layer of neonatal neurons and astrocytes, the stem cells can differentiate into all three identifiable brain cell types. Next, astrocytes from a region of the neonatal brain (hippocampus), containing a cell specific marker, GFAP+, were cocultured with NSCs. The progeny then became neurons rather than glial cells. The astrocytes produced factors that increased the rate of neuronal commitment by six times and the proliferation of neuronal progenitor cells by twofold. Both diffusing and cell surface factors from the astrocytes stimulated proliferation of the stem cell progenitors. The astrocytes obtained from the spinal cord of neonatal or adult rats showed little or no effect on either stem cell specification or proliferation indicating the regional specificity of the astocyte source. The neurons produced both possessed a nerve cell marker (MAP2ab+) and the presence of the GFP+ verified their derivation from the stem cells. Similar GFP-labeled stem cells cultured either with fibroblast cells or purified neurons did not change their rate of proliferation. These results indicate that astrocytes from the postnatal rat brain hippocampus, rather than being just supportive, can direct neurogenesis and induce proliferation of neuronal stem cells.

Another study has suggested that the ability of NSCs to form astrocytes or neuron progenitor cells may be influenced by cytokines produced following brain trauma[115]. Brain damage induced by infection or irradiation caused inflammation in the rat hippocampus. This was caused by the production of certain cytokines, interleukins IL-1, IL-6, and tumor necrosis factor from resident microglia and macrophages. Microglia normally perform in an immune capacity and produce trophic growth factors. These cytokines subsequently reduced or regulated the formation of neuron progenitor cells, but favored the continued production of astrocytes. It was also found that the inflammation could be corrected by the injection of a nonsteroid anti-inflammatory agent, indomethacin, linked with a lipopolysaccharide that may have application following irradiation of tumor cancer patients[113].

## The Migratory Potential of Neural Stem Cells

One of the characteristics of stem cells, their ability to migrate, has opened up a whole new concept for their use. The ability of neural precursor cells to migrate and to differentiate in response to developmental cues from the host organism was shown when human neural precursors were recovered from the brains of birth defect fetuses. After cultivation with growth factors (EGF and FGF), the multipotent cells or neurosphere were deposited directly into the brain (cerebral hemisphere) ventricles of embryonic mice[116]. From there the precursor cells entered the epithelium and migrated into the fore-, mid-, and hindbrain of the hosts. In the different regions, the neural precursors could be identified by human-specific DNA probes and tissue-specific antibody reactions to a human enzyme. Depending on the region, the cells differentiated into either neurons, astrocytes, or oligodendrocytes.

The migratory power of NSCs was coupled with a second characteristic, their ability to detect areas of cell damage (cited earlier)[117]. This study involved brain tumors. Intracranial brain tumors affecting the glial cells (gliomas) were experimentally produced in the frontal lobes of adult rat brains. Other more invasive tumors, a rat glioblastoma and human glioblastomas, were implanted into the brains of adult female hairless “nude” mice. The rat glioblastoma was genetically altered to express the GFP for tracking purposes. NSCs were then introduced either by injection directly into the tumor bed or by injection into normal brain tissue from the same hemisphere of the frontal lobe or into the more distant opposite hemisphere, the connecting liquid filled brain cavity (ventricle), or by injection into the circulatory system. *In vitro* culture studies were also conducted to assess the response between the tumor cells and the NSCs. The brains from the injected animals recovered after 6–21 days were submitted to a cell destruction (oncolysis) assay or sectioned into thin slices and examined by tissue assays with chemical stains or antibodies. The migratory ability of the rat glioma and glioblastoma cells was traced by the use of a transgene (lac-Z) that permitted identification of the NSCs in the brain using assays for an enzyme (B-galactosidase). They were visualized either by chemical staining or by antibodies linked to stains and fluorescent compounds. When rat glioma or glioblastoma cells were injected into the brain tumors, they infiltrated the tumor cells and surrounded the tumor mass. They even “chased down” the rapidly spreading, highly invasive tumor cells. The NSCs implanted distant from the implanted tumor, either in the same hemisphere or in the opposite side of the brain, were able to migrate through the normal tissue into the tumor cells and still express the LacZ transgene. When NSCs were introduced via the circulatory system, they also targeted the tumors, but at a lower efficiency. Human NSCs were also able to migrate from the opposite hemisphere of the mouse brain into a glioma or glioblastoma.

The murine NSCs were also genetically altered with the gene for an enzyme, cytosine deaminase (CD), a therapeutic relevant molecule for tumor control. A complementary copy (cDNA) of the gene for the CD was produced and a complex was constructed with a retrovirus vector that was used to introduce the transgene into the NSCs. The enzyme CD converts a nontoxic precursor (5FC) into a tumor-destroying drug (5-fluorouracil). This is a chemotherapeutic agent that is toxic for rapidly growing cells. The effect of NSCs transduced by the gene for the enzyme indicated that in cell cultures, the CD-NSCs grown with glioma cells caused death in the surrounding cells. Then, after inoculation into the circulatory system, the CD-NSCs found their way into the intracranial gliomas of mice and produced an 80% reduction in the tumor mass. While many questions remain, such as what causes the NSCs to home in on trauma areas, the possibility of stem cells acting as delivery agents of therapeutic compounds is apparent.

## The Retina

In lower animals such as the amphibian salamander, the regeneration of the neural retina or the lens (Wolffian regeneration) from cells in the pigmented retina had been extensively examined[118] and has been cited as a prime example of metaplasia or the now-preferred transdifferentiation. Recent experiments on the zebrafish retina, having a neuronal cell structure much like the human eye, have shown the response of resident stem cells and the further contribution of Muller glial cells during regeneration of cells of the neuronal retina[119] replacing specific cell types in the neuronal retina from resident stem cells and glial cells. The specificity of the regenerative response is very precise; the researchers found that if only rod photoreceptors are lost, only rod receptors are regenerated. The death of both rod and cone photoreceptors allows regeneration of both types of receptors and only in the damaged region. Another effect of light trauma to the retinal layer involves changes in the Muller glial cells that begin to form neuronal cell proteins. Using a transgenic zebrafish cell line that possesses GFP in its neuronal cells, they found that a portion of the glial cells also exhibited the fluorescent protein during retinal regeneration. The retinal stem cells are a good candidate for generic progenitor cells that are capable of modulation while the glial cells may transdifferentiate into neurons.

Up until now, it was thought that the mammalian retina was incapable of regenerating any new neural cell components. Recently, stem cells have been identified and isolated from the adult mouse eye[120]. These cells are restricted to the pigmented ciliary margin of the eye, a region comparable to that in the amphibian eye. When allowed to proliferate in culture, the retinal stem cells form spheroid colonies of cells that can differentiate into retinal cells, rod photoreceptors, and bipolar neurons. While in the intact retina, they are presumably inhibited from proliferation and may ultimately be the source of new cells for damaged retinas in humans.

## Stem Cells vs. Tumor Cells

Stem cells and tumor cells share fundamental biological characteristics, such as their potency for self-renewal and a multipotency for differentiating into alternate cell types. This was seen in the ability of teratomas from mouse germ cells to differentiate into normal cells and again in the formation of teratomas from human ES cells. In both the normal and abnormal states of cell proliferation, the mechanisms for controlling cell proliferation present in tumors and self-renewal in stem cells may be related. A recent investigation has proposed a link between tumor cell suppression and stem cell proliferation[121]. In mice, suppression of tumor cells is controlled by a protein called PTEN (phosphatase and tensin homologue), a lipid enzyme (phosphatase) that is a critical tumor suppressor affecting cell signaling pathways and gene transcription. Mutation of the controlling PTEN gene results in the formation of spontaneous tumors of the prostate, colon, and skin. In humans, the same gene has been noted in several malignancies of the uterus, breast, prostate, and nerve cell tumors (glioblastomas). PTEN suppresses tumor cell proliferation and possibly restores the pluripotential of cells. It has also been implicated in tumor cell invasion and metastasis. Mouse embryos carrying the homozygous mutant of the PTEN gene undergo abnormal development particularly in the central nervous system and die from abnormalities and excessive cell proliferation.

Another study on the effect of PTEN on early brain development[122] utilized gene targeting in knock out mice by inducing the mutant form of PTEN found only in embryonic neural tissues. The conditioned mice, lacking PTEN, developed large abnormal brains as a result of increased cell proliferation, in addition to very large cell size and decreased cell death. Next, the neuronal stem cells in these animals were examined to see how PTEN controls neural stem/progenitor cell proliferation. Small neurospheres or clusters of neuronal progenitor cells were isolated from the brains of the PTEN mutant mice. The researchers found that the spheres were larger in the mutants, due to an increased number of stem cells and enlarged cell size. Further studies indicated that PTEN inhibited neural cell proliferation by controlling the progression of the cell cycle. Normally, the stem cells produced specific progenitors for mature neurons, astrocytes, and oligodendrocytes. The *in vitro* culture studies showed that the PTEN deficiency increased NSC and progenitor cell proliferation and self-renewal, but did not affect the multiple lineage into the differentiated cell types. They concluded that the absence of PTEN results in increased proliferation and self-renewal of NSCs. Possibly an inactivation of PTEN could be utilized in the proliferation of NSCs therapeutically for the treatment of neurodegenerative diseases.

### *Cancer Stem Cells*

There is growing evidence that mutant stem cells may be responsible for producing malignant tumors. Cancer stem cells have appeared in some leukemia, breast, and brain cancers (reviewed by Jean Marx[123]). The native stem cells and cancer cells are both self-replicating. As in normal stem cells, cancer stem cells are the reserve cells that keep tumors proliferating. One research group (Dick et al.) has found that both normal hematopoietic stem cells and AML-leukemia causing cells shared the same pattern of cell markers, positive for a CD34 marker and negative for a CD38 marker, but neither resembled the mature, normal blood cells or malignant leukemia cells. They postulate that the mutation causing leukemia originated in normal stem cells. Another group (Weisman) thinks that gene translocation originates in normal blood stem cells that both self replicate and give rise to mature abnormal blood cells found in leukemia patients.

In a related study on breast cancer cell proliferation in an immunodeficient mouse, other investigators (M. Clarke) identified tumor stem cells from other breast tumor cells by a cell surface marker. These self-renewing cancer stem cells were isolated and used to initiate breast tumors after injections in mice, while other cancer cells from the same tumor were incapable of inducing tumors after injection. Stem cells have been isolated by P. Dirks from brain tumors as well (astrocytomas and glioblastomas) and are believed to cause the tumors. These stem cells also share markers with normal brain stem cells, but lack the markers of the more differentiated brain cells. The number of tumor stem cells seems to be related to the degree of malignancy.

## Endodermal Derivitives

### *Formation of Embryonic Liver and Pancreas*

In early embryonic development, undifferentiated endoderm gives rise to buds that are destined to form liver (hepatic) or pancreatic cells. Embryonic mesodermal endothelial cells can migrate to and surround either of the endodermal buds and deliver signals for their development into liver or pancreas. The embryonic mesoderm cells are precursors to the endothelial cells that line the blood vessels. They later differentiate into primitive blood vessel tubules.

## Embryonic Stem Cells as Blood Vessel Precursors

Cultures of mouse ES cells were targeted to form cells with a receptor (FLK1) for a vascular endothelial growth factor (VEGF) that promoted their differentiation into endothelial cells[124]. When exposed to a second growth factor, a PDGF, the same FLK1 cells can form progenitors for multipotent mesenchymal cells capable of developing blood vessel–associated cells (vascular pericytes) and blood vessel (vascular) smooth muscle tissue. These two cell types simulated blood vessel formation when cultured in a collagen gel. The potential to form similar vessels *in vivo* was tested by injection of tagged FLK1 cells into the hearts of chick embryos. After 2–3 days, the (Lac Z) tagged ES-derived cells were found in the vascular tissue of the head, heart, and mesodermal tissues of the chimeric chick embryos.

Endothelial cells are necessary not only for liver bud blood vessel formation, but also function in liver bud development. In the mouse embryo, application of a definitive antibody to a marker for embryonic endothelial cells, a platlet endothelial cell adhesion molecule (anti-PECAM), showed that the endothelial cells and the liver endoderm cells coexist before the liver bud emerges. Experiments on FLK1-mutant embryos that lack endothelial cells also showed that normal liver bud outgrowth is thwarted before movement of the mesenchyme into the region. Parallel studies on liver bud tissue *in vitro,* in the absence of endothelial cells in the mutant tissue or when they were inhibited from forming, caused a specific defect in the liver bud outgrowth[125].

## Human Embryonic Stem Derived Endothelial Cells

A human ES cell line (hES cells) obtained from the ICM of a human blastocyst became the source of vascular endothelial cells that form blood capillaries and line other blood vessels. Differentiation of the stem cells into endothelial cells and vascular structures has great potential for producing new blood vessels and inducing heart muscle cell (cardiomyocyte) regeneration[126]. The hES cells were removed from their culture on feeder cells and grown in suspension culture to form embryoid bodies (EB). The EB cells began to differentiate and expressed several endothelial cell–specific genes and also began to form vascular-like structures within the EB. The EB were then separated into cells and treated with fluorescent-labeled antibodies to the adhesion molecule (PECAM1) and the reactive cells were isolated by a cell sorter (flow cytometry). Under specific growth conditions, the cells expressed characteristics of blood vessel endothelium showing cell markers similar to endothelial cells of the umbilical cord vein and also formed a characteristic low-density lipoprotein. When these endothelial cells were seeded *in vitro* into a sponge gel, they formed capillary-like vascular structures. After transplantation of the matrix gel sponge seeded with endothelial cells into the backs of mice, the cells continued transformation into micro-blood vessels containing mouse blood cells. After 14 days, they formed long tubules resembling blood capillaries. Since human embryonic endothelial cells can be maintained in culture, they have a wide therapeutic potential for the repair of dying tissues, the implementation of vascular grafts, and the induced vascularization of regenerating tissue.

## The Embryonic Pancreas

There is a marker for early pancreatic development in the form of a transcription factor controlled by a bank of genes (Pdx1). When embryonic mouse cells of the major artery, the aorta, are combined with endodermal cells *in vitro*, the genes for the Pdx1 transcription factor are activated along with insulin production[127]. In the absence of the embryonic aorta cells, the opposite effect was found in mouse and frog embryos (*Xenopus*). If they removed the precursor cells to the aorta, the pancreatic precursor cells failed to form. The two transcription factors and the pancreatic hormone, insulin, were greatly reduced. When transgenic mice possessing the VEGF gene and promoters of the pancreas transcription factor were produced, this resulted in overactivity of the VEGF gene causing overgrowth (hyperplasia) of the pancreas primordium and overproduction of adjacent blood vessels.

The role of endothelial cells in the correction of various liver and pancreatic disorders such as lipid disorders and diabetes could be very significant for their treatment. The utilization of ES cells that could be directed into liver hepatocytes or pancreatic cells will be dependent on learning more about the inducing factors found in the embryonic endothelium[128].

### *Differentiation of Embryonic Stem Cells into Pancreas*

ES cells, being pluripotent, have the inherent ability to differentiate into any cell type that the intact embryo would normally produce. Previous examples have been both skeletal and heart muscle, blood, and brain cells. Another advantage of ES cells is that they can be manipulated into specialized tissues and organ structures. They are a promising model for future clinical treatment of diabetes through their ability to differentiate into cells that produce pancreatic hormones, including insulin, and then coincidentally develop into structures resembling normal pancreatic islets[129]. In normal embryogenesis of the pancreas, the endocrine-producing cells of the gland form an intimate complex with the neurons. Mouse ES cells in culture produced embryoid bodies after treatment with a cell division inducer (mitogen), the bFGF, and other supplements, B27 and nicotinomide. The cell populations produced were positive for nestin, a protein filament of neural precursor cells and also the cells expressing endocrine pancreatic genes. Cell division ceased and differentiation ensued after withdrawal of the mitogen. As differentiation of the ES cells progressed through five stages, a series of markers for pancreatic cells were detected. Identification of the progenitors, neurons, and insulin-positive cells was obtained with various immunocytochemical tests coupled with confocal microscopy. Concurrent with the insulin-positive cells, three additional endocrine hormones were detected: glucagon, somatostatin, and a pancreatic polypeptide. Simultaneously, formation of pancreatic-like cell clusters took place. Insulin release in response to physiological concentrations of glucose that reached a peak at stage 5 was reported. This coincided with differentiation and morphogenesis of the islet-like clusters, even though the amount of insulin produced was low. The islet-like cell clusters from the ES cells were also grafted subcutaneously into the shoulders of diabetic mice. The grafts became vascularized and remained immunoreactive to insulin at 12 days, 4 and 6 weeks after implantation. The ES-derived pancreatic cells in mice became a model for the potential source of pancreatic islets in the treatment of diabetes in humans.

Substantiation of the formation of islet-like structures from both murine and hES cells was obtained by Rajagopal et al.[130]. Furthermore, they found that the cell clusters concentrated stained fluorescent antibodies that bind to insulin as reported above. However, using techniques (RT-PCR) to detect mRNA for insulin, they were not able to detect appreciable amounts of insulin being produced in the cell clusters. The absence of insulin transcripts suggested the cells were concentrating insulin from the culture medium. Other evidence to support this interpretation came when they cultivated the cell clusters in an insulin-deficient medium and found this negated the positive fluorescent staining. While still open to further tests, they believe that insulin staining alone is not sufficient evidence for ES cell differentiation into beta cells that produce insulin.

## STEM CELL POTENTIAL

### Stem Cells in Perspective

Since the isolation of ES cells from the ICM of the mouse blastocyst embryo and the identification of separate tissue stem cells, the ability of stem cells to differentiate into multiple adult cell types, whether derived from pluripotent ES cells or multipotent stem cells from the bone marrow, brain, etc. has revamped our view of the stability of differentiated adult cells and tissues. It has challenged our concept of how stem cells differentiate, has compromised our view of how adult cells and tissues might regenerate, and projects a putative source of stem cells during normal physiological cell and tissue replication, replacement, and repair. The mouse ES cells are also a model for studies on differentiation *in vitro*. Further, the ability to manipulate the ICM of transgenic mice has enhanced the study of individual gene activity by the use of mutant genes or elimination of genes in knock out mice. Much remains to be learned from stem cell studies in mice and other animals about the enormously complex transition of stem cells, often through a hierarchy of subservient multipotent stem cells, precursor cells, and progenitor cells. The impact of cytokines and growth factors that regulate the proliferation and differentiation process are coupled with gene-controlled cell molecular signals that guide their differentiation *in vivo*. This applies both to which genes are transcribed as well as the effect of epigenetic controls on transcription. Even then, the clinical application of these findings in humans often leads to new unanticipated problems.

The relationship between dopamine-deficient neurons and the ability to control body movements is a characteristic of Parkinson's disease, where these neurons have degenerated. Early signs of treating this disease came when embryonic neural precursor cells were grafted into the brain (striatum) of a mouse animal model that had degenerated dopamine neurons[131]. The deficient neurons were replaced and afforded a partial recovery of movement. This condition can be simulated by injection of amphetamine into the brains of rat that cause them to turn aimlessly. Now, investigators[132] have harvested mouse ES cells and injected them directly into the brain-damaged rats. After 9 weeks, these ES cells developed into dopamine-producing cells, like those lacking in Parkinson patients. In high concentrations, the cells tended to form teratomas, but when diluted to about 2,000 cells/ brain, the tumors were eliminated and the ES-derived neurons were found in 56% of the host brains. A protein marker typical of the dopamine-producing neurons was also found in many of the neurons. At the same time, 40% of the survivors showed some improvement in their behavior. It remains to be seen whether manipulation of the ES cells into precursors of dopamine-producing neurons would improve the efficacy since earlier studies with neurons recovered from mouse brains (by McKay [101] and others) indicated they can be engrafted into the nervous system with the better results. Prior manipulation of ES cells into dopamine precursor neurons may be the answer. Most especially, the ability of ES cells to develop into any kind of cell (McKay) may have application in treating cardiovascular diseases, diabetes, and MS in the human.

## Stem Cells from Adult Tissues

With the discovery that stem cells residing in many adult tissues have the ability to proliferate and differentiate into their resident tissue cell types such as muscle stem cells, it became apparent that these cells could be used for the treatment of specific diseases such as Duchenne muscular dystrophy in humans. Similarly, restoration of the entire blood cell population could be obtained with hematopoietic stem cells obtained from bone marrow. It was then realized that many of these tissue-specific stem cell progenitors harbored an expanded potential, given the proper environmental cues. An elaborate procedure to purify hematopoietic stem cells by multiple animal passage through irradiated hosts was coupled with the inoculation of single, labeled hematopoietic stem cells into irradiated animal hosts[133]. These cells were reported to differentiate into both hematopoietic progenitor cells and became engrafted into epithelial cells of the liver, lung, gut, and skin where they purportedly transdifferentiated.

Potential stem cells obtained from muscle of adult mice were able to re-establish the entire hematopoietic blood cell family after irradiation. Cloned NSCs were also capable of repopulating the hematopoietic system following its destruction. In other examples, muscle stem cells could take part in muscle regeneration as well as bone healing. The presence of multipotent mesenchyme cells in human adipose tissue may provide an alternate source of cells to NSCs from the bone marrow.

Another notable aspect of specific adult stem cells is their apparent ability to be reprogrammed. NSCs isolated from adult mice and reinoculated into early embryos reverted to a pluripotency level when their progeny were found in liver, spinal cord, stomach, and kidney. Furthermore, specialized rat precursor cells for oligodendrocytes were capable of being reversed into NSCs, then redirected into precursors for alternate pathways leading to neurons or astrocytes. However, it is premature to abandon the concept that stem cells found in specific tissues are developmentally restricted, even though they may appear to have a multipotential within their own compartment. Failure to isolate individual stem cells or to rule out contamination by other endogenous stem cells must be critically evaluated. The ability of stem cells to expand their horizons and transdifferentiate into diverse tissue types is still subject to scrutiny. Several reports cited earlier have failed to confirm transdifferentiation of bone marrow stem cells or blood cell stem cells to brain cells.

Most adult stem cells such as bone marrow stem cells have a multipotential limited to form diverse kinds of blood cells. Reports of neural cells that transdifferentiate into blood[105] or NSCs labeled with the enzyme beta-galactosidase and cultured with embryoid bodies[106] developed beta-galactosidase–labeled muscle cells that may be the result of different mechanisms. These events have been offered as examples of transdifferentiation. However, the stem cells might have been contaminated with muscle cells in the latter example, or mutations could have occurred or cell fusion might have been responsible[134]. Recent experiments would favor the last possibility. Cultured adult mouse bone marrow cells resistant to puromycin were harvested and marked by a green protein tracer together with ES cells that had no fluorescent marker[99]. This produced hybrid cells with enlarged nuclei. These fluorescent puromycin-resistant cells appeared and acted like ES cells, but became heart muscle cells. Further examination showed the cells had multiple sex chromosomes, including a male Y chromosome from the ES cells and multiple copies of chromosomal DNA. The adult cells formed a hybrid and fused with the more dominant ES cells. A similar investigation[135] on adult mouse brain stem cells containing a transgenic marker indicated that they underwent spontaneous fusion when cultured with ES cells that expressed a protein called Oct 4. The hybrid cells contained both cell markers[98]. This suggests that the observed plasticity of many adult stem cells may be the result of a change in the developmental fate (transdetermination) subsequent to cell fusion rather than expanded nuclear reprogramming.

Thus, putative transdifferentiation experiments will require detailed genetic analysis since the observed *in vitro* cell fusion may have been favored by the extended culture and does not address what happens *in vivo*[134] and caution an interpretation of similar apparent transdifferentiation experiments. Still, if bone marrow cells repair heart damage in rodents, the cells are potentially useful clinically. As Wang's study[88] of cell fusion of bone marrow stem cells and liver hepatocytes suggests, it is doubtful that bone marrow hematopoietic stem cells have little differentiation potential other than their blood cell lineage. While the donor bone marrow cells can be of use therapeutically, it necessitates increasing the efficiency of cell fusion (rather than expanding stem cell progenitors of hepatocytes).

Functional transdifferentiation of stem cells needs to be addressed as a concept before its implication in a therapeutic regimen. Standards for this have been proposed[136]. First, the isolated stem cells should be transplanted without intervening modifications from culture manipulations. Second, the stem cells should provide a substantial regeneration of the target tissues. Third, transdifferentiation of the stem cells needs to be substantiated for morphological, molecular, and functional properties such as muscle cells demonstrating contractions driven by actin and related molecular events. Lastly, conversion of stem cell progeny should be capable without prior organ damage.

The cell migratory capability observed in muscle, bone marrow, and neural tissues after their dissemination through the circulatory system, the brain chambers, or by direct inoculation into tissues has expanded their therapeutic application. These range from correction of nerve cell damage to an avenue for treating cancer sites. The most exciting aspect is to combine this capability with transgenic stem cells as a means of targeting specific cells in distress.

Adult stem cells have certain other restrictions. Ideally, stem cell therapy would be most successful if they are derived from a single patient and their progeny returned to the same individual, since this would reduce the problem of the immune response and rejection of the engrafted cells. There are other limitations. Not all adult tissues have yielded stem cells, but that may change as research continues. Second, while many stem cells have a multipotential that allows limited deviations, such as the ability of hematopoietic cells to form not only diverse types of blood cells but other types of cells, the ability to direct and control changes in the stem cell pathways are not always reliable. The infusion of bone marrow cells with their stem cells into human diseased hearts in clinical trails in Germany has been partially successful in improving heart pumping function. It has not been clear that the bone marrow cells form new cardiomyocytes and in some cases serious arrhythmia have occurred. Another shortcoming is the small numbers of adult stem cells available for recovery in order to grow sufficient numbers for cell culture along with limits on their cell line longevity. Many tissue stem cells are notoriously difficult to identify *in vivo*. The amount of tissue available may be limited when we are dealing with humans. This is particularly critical if these cells are to be recovered from a patient to avoid the immune response. Coupled with this is the fact that the number of stem cells available from an individual decreases with age.

## Embryo-Derived Stem Cells

Except for the presence of naturally occurring somatic stem cell systems, such as hematopoietic stem cells in the adult, stem cells derived from embryonic ICMs have some distinct advantages for their utilization therapeutically. First, they are easier to identify, isolate, and use as established stem cell lines. Such stem cell lines can be maintained in culture indefinitely, serving as an instant reservoir to precursor cells when needed. Since they are pluripotent, they can be manipulated into any derivative of the three primary embryonic germ layers, such as described in the formation of precursors to pancreatic cells in the mouse. Moreover, they are less prone to elicit an immunological rejection during therapeutic trials. However, many more studies on human-derived ES cell lines are needed before they become useful in the clinical setting.

When Thompson et al.[31] isolated stem cells from human blastocyst embryos and established several "immortal" cell lines, it opened the possibility for manipulating human totipotent cells in the treatment of many human diseases and deficiencies. The production of a similar human stem cell line, EG from PGC, provided an alternate opportunity. Being embryonic, these lines have a much longer lifespan than similar adult stem cells. Projected other uses for the cells range from a means to study human embryonic developmental processes *in vitro*, the treatment of birth defects, and infertility and pregnancy loss. As more is learned about the events that control embryonic differentiation, the same information can be applied to understanding how to direct similar differentiation steps in stem cells toward a specific cell type or tissue. Being pluripotent, they would provide directed differentiation into specific cell types for injection or engraftment in the clinical treatment of diseases or for screening the therapeutic effects of drugs on specific cells, reducing the need for animal testing. The production of a bank of human cell lines, each with its own transplantation signature, would provide a reserve of embryonic cell lines with known transplantation compatibilities to moderate rejection by the immune system. These lines could be engineered with transgenes, permit gene targeting in the event of defective genes, or the production of ES lines containing the genome of the transplant recipient[32].

## Stem Cells and Somatic Cell Nuclear Transplantation (SCNT)

An alternative approach to using embryos for stem cells is that of *nuclear* cloning. In the contemporary cloning procedure, an animal clone can result from the transplant of a nucleus from an embryo into an enucleated egg cell or the transfer of a somatic cell nucleus from a specialized adult differentiated cell into an egg cell lacking a nucleus. In the case of therapeutic cloning, the cloned cell has the same genome as the parent, but part of the cytoplasm comes from the donor cell and part from the oocyte host cell. Most experimental procedures now utilize cell fusion usually obtained by an electrical stimulus. With the production of Dolly, a lamb cloned from the nucleus of an adult cell introduced into the oocyte, the ability to clone animals for agriculture became a reality. Subsequent use of the technique led to the formation of the first transgenic clone, where genes for synthesizing human proteins were introduced into an animal cell that created "new genomes in medicine as well as agriculture"[13].

Recent proposals[137] have described the basis for "therapeutic cloning", whereby nuclei from human adult cells (hSCNT) are transferred into oocytes to produce a human blastocyst embryo that would be used to establish a unique pluripotent ES cell line. Given this intent, it is important to distinguish hSCNT from the potential of "cloning" that is applicable in domestic animal procedures. After SCNT, the activated donor egg cell would develop up to the blastocyst stage when cells of the ICM could be used to establish an individualized ES cell line as a reservoir for tailored adult cells. Direction of these stem cells into nerve cells, for example, could provide a pool of cells for therapeutic application that would be subject to a reduced immune response. A further use would be to understand human genetic-controlled diseases rather than a source of stem cells. As found in animal cloning, this procedure could be subject to many pitfalls, such as very low efficiency of blastocyst production, an adequate measure of a normal genome, abnormal chromosomes, aberrant molecular pathways, etc. The resultant embryo, formed experimentally, without normal fertilization, could provide the source of stem cells separate from embryos left over after *in vitro* fertilization, even though the former procedure is the basis for reproductive cloning.

There first appeared to be inherent limitations on the success of nuclear cloning in primates (monkeys and humans). Following SCNT in rhesus monkeys[138], it was found that the cells' division spindle, the framework for guidance tracks of chromosomes during cell division, became disorganized. Many of the cells end up with unequal numbers of chromosomes. Two key proteins, NaMa and HSET, normally found near the chromosomes of unfertilized eggs, were missing after removal of the nucleus from the oocyte. Even though the technology for this procedure exists, and such problems may be overcome, many reputable scientists feel that research on human cloning should be banned as it is in the U.S. and many other countries (see later).

In a major scientific endeavor, scientists in South Korea have succeeded in producing hES cells after cloning somatic human ovarian follicle cells[139]. A hES cell line was initiated by nuclear transfer (SCNT) of human follicle (cumulus) cells that surround, protect, and nurture the oocyte in the ovary. These cells, previously found successful in cloning mice and cattle, were the source of nuclei that were then inserted into anuclear human oocytes. After simulating egg activation and inducing cell division, 28% of the embryos developed into blastocysts. Isolation of stem cells from the blastocyst ICM provided a cloned human stem cell line (SCNT-hES-1). These cells retained their normal karyotype for 70 generations and were capable of forming embryoid bodies *in vitro*. Evidence for the cells pluripotency was obtained when the latter were implanted into immunodeficient mice. There the implants formed diverse differentiated cells from all three embryonic germ layers, such as retinal and bone cells. Although various analyses of imprinted genes indicated that the stem cells had a dual maternal/paternal origin, it could not be absolutely certain that they were not produced by parthenogenesis, a feat successful only in monkey thus far[140]. The ramifications of reproductive cloning and the production of humans by this procedure presently banned in most countries is yet to be determined.

A still different methodology for producing stem cells has its origin in the process of parthenogenesis, i.e., development of the oocyte by activation without biparental contribution of chromosomes. Some animals, e.g., insects, frogs, and snakes, have evolved this natural form of development. Artificial egg activation in other forms is also possible. Artificially activated mouse eggs (parthenotes) suggested an alternative to SCNT and provided the potential for producing ES cells. In mice, cattle, and primates, these embryos do not support full-term fetal development. However, preimplantation stages appear to be normal. Recently, monkey parthenotes were produced[141] that yielded stem cells capable of forming into all three embryonic germ layers. These primate eggs (oocytes) were recovered and activated parthenogenetically. In sperm-activated oocytes, the egg nucleus divides once reducing the chromosomal number by half. By suppressing the usual nuclear division of the artificially activated oocyte, the chromosomal number was doubled back to the normal, but now identical chromosomal (alleles) occurred. Under culture, the cells divided and developed to the blastocyst stage. Subsequently, cells from the ICM of a monkey blastocyst produced an ES cell line. *In vitro*, some of these ES cells formed dopamine-producing neurons. Other cell types, such as beating cardiocytes, smooth muscle cells, and ciliated epithelial cells were found. Injection of the cells into adult hosts also produced teratomas with highly differentiated tissues. If this procedure is found successful in human eggs, it would serve as an alternative source of ES cells. Additionally, for females at least, it could provide a source of immunological compatible cells.

Still another avenue has opened with the formation of oocytes from ES cells in culture. Embryonic mouse stem cells are capable of spontaneously forming germ cells (oocytes) in culture[142]. Mouse ES cells were transfected with a germ line–specific reporter gene, Oct 4, specific for early stages of germ cells and coupled to GFP. The cells were first found to be expressed in the developing germ cells of transgenic mice that were derived from ES cells. Some of the same transfected ES cells were placed in culture. After 8 days, up to 40% of the cells were positive and some formed follicle-like cell clusters around oocytes (the latter detected by the presence of unique zp2 and zp3 proteins). The follicle cells normally nurture the oocyte in the intact ovary. These cells also produced estradiol, an estrogen hormone found in the ovarian follicle cells. After 40 days, groups of sixteen-cell morula-like embryos formed and expressed the Oct 4 protein in the nucleus. A few blastocyst-like stages formed and since they were not fertilized would be considered to be parthenotes. Although the cells have not been shown to be fertilizable, the potential therapeutic application in humans would open a whole new source of stem cells.

## Embryonic Stem Cell Application in the Human

### *The Nature of the Human Embryo*

Throughout the animal kingdom, the union of a sperm with an egg initiates a new lifeform of a particular species with the inherent capability of developmental change leading only to the advanced stage of that individual species. The merging of the chromosomes and establishment of a new genome triggers a series of events that are preparatory to the identity of the species, but all species share common genetic events such as chromosome duplication, cell division, and differentiation. These early embryos develop into a blastula or the equivalent mammalian blastocyst by default. The early events are often under the direction of the maternal genome. It is only in the blastocyst that major changes in differentiation, now driven by the (combined) embryonic genome, begin to unfold. The human blastocyst is a pivotal stage since the human potential is not realized unless implantation into the uterine wall takes place. This early primordial embryo ceases to develop further *in vitro*; failure to implant *in vivo* leads to degeneration and loss. Many developmental and reproductive biologists support the concept that the developmental formation of a human embryo does not occur until implantation is accomplished.

### *The Status of the Pre-Embryo*

Once the human oocyte is fertilized and activated, the early stages of development produce the blastocyst whose mission is to prepare for implantation. Scholars of human embryology[1,2] recognize these stages as preimplantation embryos. Gradually, the term pre-embryo was proposed to describe the initial postfertilization phases of mammalian and human development[5] and McLaren proposes that the aggregate of precursor cells is a developing entity, a pre-embryonic phase[143,144]. Preimplantation embryonic stages were considered a developing (potential) form of human life[145] by an NIH panel. Others disagreed with the designation of pre-embryos as being ill defined, inaccurate, unjustified, and equivocal[2]. Perhaps the choice of another term, such as primordial embryo, etc. would have been more appropriate. Gradual use and application of the term pre-embryo[146] has resulted in the usage by some embryologists[147] to designate the early pre-embryonic human stages as being from the zygote through the blastocyst stage.

The blastocyst stage has two basic functions: first to complete a successful implantation of the formative ICM cells with its protective membranes and then to establish a primary axis for each potential embryo(s). Those blastocysts that do not implant successfully will die and be sloughed off. All single or twin fertilized eggs are also subject to loss by normal attrition. Estimates of “lost” fertilized eggs that fail to implant range from 30–70% of blastocyst embryos, many of which are defective and are rejected[148]. Information from identical twinning in humans indicates that each of the early cleavage cells have the potential to form a human embryo and fetus provided the ultimate blastocyst stage survives to implant in the uterine wall. Identical twining is possible in early cleavage up until the eight-cell stage, when the capability of individual blastomeres is restricted . Later, the appearance of dual ICMs by the $4^{th}$ to $5^{th}$ day can lead to identical twins when two or more prospective embryonic axes may be established (the bilaminar disc stage) of the preimplantation blastocyst. More rarely, twinning can also occur in the 2nd week (postimplantation) by duplication of the embryonic disc[2].

If implantation of the blastocyst is successful, the process of gastrulation is initiated when one or more primary axes form. It has been proposed by some that this is the point of *individuation,* the time when the single (or plural) embryonic axis (primitive streak) is established. By this view, the early conceptus (trophoblast + ICM), being totipotent, may become only one life or it may develop into a hydatiform mole or it may form twins[149]. In this context, an individual human embryo is established only when further division of the implanted embryo cannot be done without causing harm to the embryo[150]. Individuation would coincide with period of development when the embryo assumes the moral status of a human person[151]. The human embryo research panel at the NIH[145] stated that the preimplantation embryo is a developing form of human life with a moral status, but does not have the same moral status of a person because of the absence of developmental individuation. On a timeline of development, the process of individuation would conform to the 2nd week, just after implantation. The use of twinning as a determination point is somewhat ambiguous since dual ICMs leading to identical twins can form at the preimplantation bilaminar disc stage (about 4–5 days) as well as later. In this context, the definitive point would seem to be when the final embryonic axis(es) form the primitive streak(s) that appears in the 2nd week between 12 and 17 days. It is important to note that for the conceptus to produce a human embryo, both implantation and primitive streak formation are necessary.

The use of human embryonic and fetal cells for research or clinical use has raised many ethical, moral, and legal questions and is further complicated by political influences. The use of human pluripotent stem cells for research is permitted by NIH guidelines since they are not in themselves embryos[152]. The principal objections have been on the use of directed abortions of embryos and fetuses based on religious or ethical concerns about the purposeful destruction of human life. The same objections by the Catholic Church have been extended to the use of frozen and stored early cleavage embryos left over from *in vitro* fertilization and would also apply to embryos produced for therapeutic cloning[153,154]. The Vatican position is to ban any use of stem cells taken from human embryos since it holds that the beginning of human life starts with conception.

For some, the crux of the matter regarding the use of the blastocyst embryo for stem cells centers on the determination of when an embryo becomes a human person. The pre-embryo is viewed as a developing entity that has a moral status with the potential to form a human embryo. McCormick[155] argues that “because the pre-embryo does have intrinsic potential” as defined, it “should be treated as a person”, although there may be acceptable conditions such as genetic abnormality, rape, etc. that may terminate its existence. Others believe that the fertilized egg and succeeding developmental stages, while having a moral value as a potential human person, does not equal that of an advanced embryo, fetus, or newborn. Some hold that the moral status of a human embryo is related to individuation, when the primitive streak is formed. Thus the use of cells from the ICM of human blastocysts and the potential benefit of the derived cell lines in the treatment of human diseases and maladies must be weighed against the philosophical determination of when a human being arises. The scientific use of hES cells, or the derivation of new stem cell lines, is subject not only to political, ethical, and moral considerations of our culture, but also religious convictions such as the determination and protection of a human entity that are concerns for many individuals. These are philosophical issues concerned with the beginning of human life and religious interpretations of the spiritual being. Most will agree that the “pre-embryo”, the blastocyst, or the preimplantation embryo all have the potential to become a human being. It seems incongruous not to consider dividing cells as an embryo since it is the initial period of mammalian or human development. The final determination of when a human embryo or person appears during development is a philosophical interpretation of a developmental continuum from the time of conception to postimplantation or any stage in between (or later). This is still not clear to many and must be decided by the dictates of one’s religious conviction or by each individual’s code of ethics. This must be weighed against the potential benefit of ES cells for curing disease and saving lives. The lawful use of ES cell lines obtained directly from early embryos while constrained, is extant.

While some of the ES lines were derived from aborted fetuses, for the most part the embryos that produced the ES cells were obtained from unused frozen embryos left over following *in vitro* fertilization and donated by couples who had provided them. The unused early cleavage cells are routinely frozen indefinitely. No longer needed, they remain frozen or are discarded after 5 years in storage. Such frozen embryos recovered are then cultured to the blastocyst stage and, unless they are implanted into the uterus, they cease development and thus many believe their use is justified. At this point, cells removed from the ICM are used to establish embryonic pluripotent stem cell lines. These ICM cells are no longer considered totipotent, i.e., capable of forming a whole embryo, nor can they develop into one after implantation. Both the ICM and the accompanying trophoblast layer, that directs and nurtures the ICM, are needed for continuing development into the human embryo following uterine implantation in the normal course of events.

ES cells from the blastocyst remain as the cells having the greatest application for the treatment of human diseases and maladies such as spinal cord injuries, Parkinson's disease, Huntington's disease, Alzheimer's disease, and the treatment of muscular dystrophy, diabetes, heart diseases, and forms of arthritis. In addition to their versatility, the difficulty in recovering sufficient stem cells from human brain or muscle tissues may mitigate the use of ES cells. ES cells can be manipulated with greater certainty into specific cell lineages, i.e., nervous tissue, pancreas, and heart muscle, etc. particularly since they have the ability to be targeted toward their differentiation goal. There is good evidence that such differentiated cells may be organized into specialized tissue structures such as pancreatic islets. Moreover, being embryonic, they can be perpetuated as cells with a normal genotype and can be customized to reduce immune rejection by the host.

The potential of generating parthenogenetic human blastocysts from unfertilized oocytes, if successful, offers another possible alternate source of ES cells. Since the living organisms produced by this procedure are not derived from both gametes by the process of fertilization, its application in humans would differ from taking ES cells from the ICM of leftover frozen embryos. Their use might have certain side effects such chromosomal defects, unmasking recessive genes, etc.

The source of stem cells from an embryo resulting from nuclear transplantation, SCNT into oocytes, or therapeutic cloning raises still another issue. It is important to distinguish this procedure from the objectives in reproductive cloning. While experimentally produced, an embryo (blastocyst) with human potential is the result. The resultant ES cell lines from such "constructed" embryos are not totipotent. Now that SCNT has been demonstrated as a viable option in humans, the issue would appear to be their intended use, whether such artificial blastocysts should be used as a source of stem cell lines or whether they are produced with the intent of making an identical human clone[156]. Second, given legitimate scientific intent for therapeutic purposes, the decision would be whether they would be preferred to the destruction of unused preimplantation embryos. Much more significantly would be their potential misuse as cloned preimplantation embryos.

Obviously, these objections on the use of human embryo–derived stem cells do not affect stem cell research on cells recovered from adults or birth tissues. Recently, stem cells were recovered directly from umbilical cords (Sanberg, paper presented at AAAS meeting, San Francisco, 2001) for the treatment of strokes. Presumably fetal in character, their exact identity and full potential remains to be seen. Related studies on rats have indicated that implantation of neuroepithelial stem cells into the brains of rats with induced occlusion of the cerebral artery produced a resolution of stroke dysfunction[157].

Another possibility would be the recovery of adult stem cells from human organ donors in the event of unanticipated death. The recent recovery of retinal stem cells that proliferate once they are released from the retina could be used for treatment of damaged or diseased retinas[120]. The time limits on the availability of these cells restricts them as a continuous source. The advances in the use of bone marrow stem cells, muscle stem cells, mesenchymal stem cells, and organ stem cells have already shown their application clinically.

## *Governmental Policies on the Use of Human Embryonic Stem Cells*

The derivation and use of human stem cells from surplus embryos is permitted in the following countries as of 2004: Australia, Belgium, Canada, Finland, Sweden, Denmark, Netherlands, Spain, Greece, United Kingdom, Israel, India, Singapore and Japan, China, Iran and North and South Korea. Some countries allow only the use of existing human stem cells for research such as Germany and the United States. Only a few governments such as the United Kingdom, Belgium. Israel, Singapore, China have condoned therapeutic cloning. Other countries such as Switzerland, Ireland, Austria, France, Poland, Italy and Mexico do not allow embryo research. It is not suprising that the Ministers of the European Union have not been able to resolve differences and failed to reach an agreement on stem cell research as of mid-2004.

German Embryo Protection law prohibits using the human embryo for any purpose other than enhancing its purposeful development in the uterus of the same individual. Recently, the German Parliament ruled on stem cell importation. It still banned the general import of hES cells, but provided loopholes with many restrictions that allow importation of ES cell lines established before the draft law was passed. In 2001, the British Parliament established rules that would permit the derivation and use of stem cells from human embryos as well as research on therapeutic cloning and in 2002, the U.K. Medical Research Council announced the establishment of a hES cell bank[159] British scientists have just been licensed in 2004 by the Human Fertilization and Embryology.

Authority to perform human therapeutic cloning (SCNT).. In Australia, as of 2002, the Parliament approved the use of established hES cell lines and permission to derive new cell lines obtained from embryos left over from *in vitro* fertilization. Furthermore, the government will invest in a new Center for Stem Cells and Tissue Repair at Monash University. They hope to generate new ES and adult stem cell lines, as well as direct stem cells into specific tissues for the treatment of disease. The government stopped short of permitting therapeutic cloning.

In the U.S., a joint report of the American Association for the Advancement of Science and the Institute for Civil Society[37] recommended both public and private support for research on human stem cells from embryos, the fetus, or adult, but withheld recommending the use of public funds, for the time, for further ES and EG cell isolations. It did favor the isolation of ES cells from blastocyst embryos remaining after *in vitro* fertilization, but only after the donors voluntarily consent for their use in research. A National Bioethics Advisory Commission[160] further recommended federal funding for both ES cell line derivation and the use of established ES cell lines. The NIH, following the recommendations from the National Bioethics Advisory Commission, in 2000 proposed research on stem cell lines and their continued derivation from embryos obtained (only) from fertility clinics. However, in late 2001, President Bush allowed federally supported research on some 60 lines already established ES cells, but prevented further research that destroys embryos. In reality only eleven of these lines have been available for use[161]. Later, the National Academy of Sciences submitted a report stating that far more cell lines are needed for research and endorsed therapeutic cloning for producing genetically compatible cells. In 2002, California permitted the derivation and use of human embryo stem cells, human embryo germ cells, adult stem cells, and embryos from SCNT, subject to institutional review boards, but it banned reproductive cloning[162]. In the meantime, privately funded research continues, such as the M.D. Fox Foundation for Parkinson's research on dopamine-producing neurons from stem cells.

## Standards for Human Stem Cells

A group of leading molecular biologists who currently utilize both animal and human stem cells (HESC) have proposed the establishment of a hES cell repository in the U.S. parallel to the Stem Cell Bank being established in the U.K.[163]. Such a reservoir would collect all the current and future HESC lines for distribution to investigators. Both the data obtained for each line submitted and their distribution would be rigidly controlled by a panel of scientists who possessed expertise in molecular embryology, data analysis, and bioinformatics. Furthermore, the registry would contain a web database that would collect and disseminate information related to stem cell characteristics, assays for assessing the lineage of stem cell differentiation, cultivation, etc. Finally a baseline of changes in normal human embryonic development would be used to monitor, predict, and measure differentiation, a necessary prerequisite for stem cells in regenerative medicine.

### Guidelines and Regulations for Human Stem Cell Research

The legitimate use of embryo-derived stem cells, regardless of their source or methodology, is subject to input and review from numerous viewpoints, ethical, religious, societal, and political. If a consensus for a national policy can be reached, as proposed in the report of the American Association for the Advancement of Science and by the Institute for Civil Society[37], the agreement should be reviewed according to established regulations and safeguards. One rule would avoid and deny having embryos created expressly for the purpose of providing cells so that only unused cells from *in vitro* fertilization would be made available. Another regulation would require the informed consent of both parents with no compensation permitted for their donation.

While guidelines regarding the need for informed consent for the use of discarded embryos from *in vitro* fertilization have been proposed, the same consent has not been extended to gamete donors to egg and sperm banks. The donation of oocytes or sperm for infertility does not necessarily extend to permission for their use in embryo or stem cell research. Consequently, a group of ethicists and scientists have proposed[164] that any donor of oocytes or sperm should also include their consent of the potential use to produce embryos for future research.

All of the ethical reports and recent scientific reviews by Di Berardino[13], McKinnell and Di Berardino[15], and Solter and Gearhart[137] agree that ES cells should not be utilized to clone human beings. In 2003, the U.S. House of Representatives approved the Human Cloning Prohibition Act of 2003 that bans research on human reproductive cloning and SCNT. Alternatively, the U.S. Senate is considering a bill that would ban human reproductive cloning, but permit nuclear transplantation under strict guidelines. The United Nations considered a proposal to ban human therapeutic cloning globally in 2003 that failed to pass. In June, 2004, a conference on human cloning was held at the United Nations that included scientists from four continents but U.N deliberations were deferred again until October, 2004..

A medical ethics panel sponsored by the Johns Hopkins University charged with studying the ethical issues using human ES cells (2003) has recommended that earlier human lines not be used for clinical research since they might contain viruses derived from the mouse feeder cell lines. Newer procedures now allow any new human embryo stem cells (still banned by the U.S. government) to be formed without the use of feeder mouse cells.

## BIOSKETCH

**Kenyon Tweedell** is a retired professor of embryology. His former research interests were on regeneration of marine invertebrates (Coelenterates) and on egg production and spawning in a marine worm (Polychaeta). His major effort has been a life-long study on the origin, characteristics, and transmission of a kidney tumor (Lucke renal carcinoma) of the common leopard frog.
